# Baf60b-mediated ATM-p53 activation blocks cell identity conversion by sensing chromatin opening

**DOI:** 10.1038/cr.2017.36

**Published:** 2017-03-17

**Authors:** Shuyi Ji, Linying Zhu, Yimeng Gao, Xiaoran Zhang, Yupeng Yan, Jin Cen, Rongxia Li, Rong Zeng, Lujian Liao, Chunhui Hou, Yawei Gao, Shaorong Gao, Gang Wei, Lijian Hui

**Affiliations:** 1 State Key Laboratory of Cell Biology, Institute of Biochemistry and Cell Biology, Shanghai Institutes for Biological Sciences, Chinese Academy of Sciences, Shanghai 200031, China; 2 CAS Key Laboratory of Computational Biology, CAS-MPG Partner Institute for Computational Biology, Shanghai Institutes for Biological Sciences, Chinese Academy of Sciences, Shanghai 200031, China; 3 Key Laboratory of Systems Biology, Institute of Biochemistry and Cell Biology, Shanghai Institutes for Biological Sciences, Chinese Academy of Sciences, Shanghai 200031, China; 4 Shanghai Key Laboratory of Regulatory Biology, School of Life Sciences, East China Normal University, Shanghai 200241, China; 5 Department of Biology, South University of Science and Technology of China, Shenzhen, Guangdong 518055, China; 6 Clinical and Translational Research Center of Shanghai First Maternity and Infant Hospital, Shanghai Key Laboratory of Signaling and Disease Research, School of Life Sciences and Technology, Tongji University, Shanghai 200092, China; 7 School of Life Science and Technology, Shanghai Tech University, 100 Haike Road, Shanghai 201210, China

**Keywords:** Baf60b, ATM, p53, chromatin remodeling, lineage conversion, hepatic conversion

## Abstract

Lineage conversion by expression of lineage-specific transcription factors is a process of epigenetic remodeling that has low efficiency. The mechanism by which a cell resists lineage conversion is largely unknown. Using hepatic-specific transcription factors Foxa3, Hnf1α and Gata4 (3TF) to induce hepatic conversion in mouse fibroblasts, we showed that 3TF induced strong activation of the ATM-p53 pathway, which led to proliferation arrest and cell death, and it further prevented hepatic conversion. Notably, ATM activation, independent of DNA damage, responded to chromatin opening during hepatic conversion. By characterizing the early molecular events during hepatic conversion, we found that Baf60b, a member of the SWI/SNF chromatin remodeling complex, links chromatin opening to ATM activation by facilitating ATM recruitment to the open chromatin regions of a panel of hepatic gene loci. These findings shed light on cellular responses to lineage conversion by revealing a function of the ATM-p53 pathway in sensing chromatin opening.

## Introduction

In mammals, the identity of a differentiated cell, shaped by epigenetic and extracellular cues, is stably maintained for tissue homeostasis^1,2,3^. During development and tissue regeneration, stem cells are committed to their differentiated identity. The stability of the differentiated identity of cells is essential for the growth, survival and perpetuation of all multicellular organisms. Recent findings have demonstrated remarkable plasticity of cell identity in mammals, e.g., *in vitro* lineage conversion induced by forced expression of lineage-specific transcription factors^4,5,6,7,8^. Reprogramming of somatic cells to induced pluripotent stem (iPS) cells was achieved by the ectopic expression of Oct4, Sox2, Klf4 and c-Myc. The use of lineage-specific transcription factors was also applied to the induction of neuronal cells, cardiomyocyte-like cells and hepatocyte-like cells^9,10,11 12,13^. Because the culture medium conditions are well defined in these experimental systems, cell identity conversion thus shown is mainly controlled by the network of lineage-specific transcription factors. In addition, cell identity conversion induced by transcription factor demonstrates that the epigenetic modifications of a differentiated cell are plastic and subjected to reprogramming.

Notably, *in vitro* lineage conversion is often a low-efficiency process. It was proposed that there is a “barrier” against lineage conversion, which was largely discussed at the epigenetic level^4,5,6,7,8^. However, the molecular basis of the “barrier” remains largely elusive. Specifically, given the importance to maintain cell identity and the plasticity of epigenetic modifications, it is interesting to ask whether there is an essential cellular mechanism beyond the epigenetic “barrier” that senses cell identity change and consequently blocks the process^12,14^. We approached this question by characterizing Foxa3, Hnf1α and Gata4 (3TF)-induced hepatic conversion in mouse fibroblasts^12^.

## Results

### Transcription factor-induced ATM and p53 activation impedes hepatic lineage conversion

**Figure 1:**
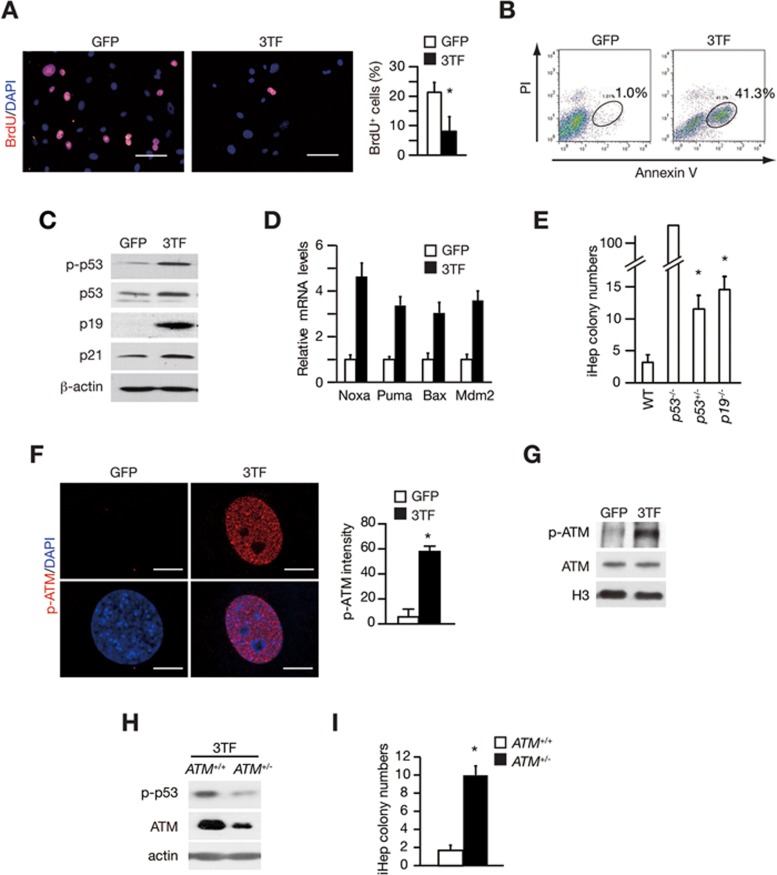
ATM and p53 impede hepatic conversion. **(A)** 3TF-induced proliferation arrest in wild-type (WT) tail-tip fibroblasts (TTFs) was determined by BrdU incorporation and staining at day 3. GFP expression vector was used as a control. BrdU-positive cells were quantified, *n* = 6 fields. Scale bars, 100 μm. **(B)** 3TF-induced cell death was analyzed by Annexin V and propidium iodide (PI) staining at day 6. **(C)** Levels of phosphorylated p53 (p-p53, Ser15), p19 and p21 were characterized by western blot analyses. **(D)** mRNA levels of p53 target genes were quantified by qRT-PCR. **(E)** iHep cells were generated from WT, *p53*^−^^/−^, *p53*^+/−^ and *p19*^−/−^ TTFs by 3TF transduction. iHep colonies were quantified 8 days after 3TF transduction, *n* = 4 independent experiments. **(F)** Elevated level of ATM phosphorylation at Ser1981 (p-ATM) was determined by immunofluorescence staining 48 h after 3TF transduction. The fluorescent intensity was quantified by LAS AF Lite software. Analyzed cell numbers: *n* = 32 for GFP group and *n* = 33 for 3TF group. Scale bars, 10 μm. **(G**-**H)** p-ATM **(G)** and p-p53 **(H)** levels were analyzed by the western blot assay in WT TTFs **(G)** or *ATM*^+/+^ and *ATM*^+/−^ **(H)** TTFs 48 h after 3TF transduction. **(I)** Quantification of iHep colonies generated from *ATM*^+/+^ and *ATM*^+/−^ TTFs. *n* = 3 for each group. Error bars indicate SD. ^*^P< 0.05, Student's *t*-test.

Wild-type (WT) tail-tip fibroblasts (TTFs) underwent a prominent proliferation arrest and cell death after 3TF transduction, which largely restrained hepatic conversion (Figure 1A and 1B and Supplementary information, Figure S1A). Our previous study showed that p19Arf inactivation facilitates induced hepatic (iHep) cell formation^12,14^. Because p19Arf is a key regulator of the p53 pathway^15,16^, we asked whether p53 acted as a roadblock to hepatic conversion. As determined by western blot analysis, we found that phosphorylated p53 (p-p53) and total p53 protein levels were increased after 3TF transduction (Figure 1C). In addition, expression levels of p53 target genes, including *p19Arf*, *p21*, *Noxa*, *Puma*, *Bax* and *Mdm2*^15,16^, were induced (Figure 1C and 1D), suggesting that the p53 pathway is activated. Importantly, upon deletion of p53, 3TF-induced proliferation arrest and cell death were reversed (Supplementary information, Figure S1B and S1C). Although only 2-3 iHep colonies were obtained from 10^5^ WT cells, iHep formation was drastically increased in p53-knockout cells (Figure 1E and Supplementary information, Figure S1D and S1E). Moreover, the generation of iHep cells was improved by p53 inactivation using *p53*^+/−^ heterozygous cells (Figure 1E) or short hairpin RNA (shRNA)-mediated p53 silencing (Supplementary information, Figure S1F and S1G). These results showed that p53 is a barrier against hepatic conversion.

Interestingly, p53 has been previously shown to limit conversions between cell identities, including iPS generation^17,18,19,20,21^ and oncogenic transformation^22,23^. We next characterized the mechanism by which p53 is activated during hepatic conversion. We did not find changes in c-Myc and Ras expression (Supplementary information, Figure S2A), Ras GTPase activity (Supplementary information, Figure S2B), p38α phosphorylation (Supplementary information, Figure S2C and S2D) or reactive oxygen species levels (Supplementary information, Figure S2E), which are major causes of p53 activation^15,16^. In contrast, extensive ATM phosphorylation was observed after 3TF transduction (Figure 1F and 1G and Supplementary information, Figure S3A). Because ATM deletion was toxic to TTFs (data not shown), we used *ATM*^+/−^ cells to characterize the function of ATM in hepatic conversion. *ATM*^+/−^ cells showed attenuated p53 phosphorylation (Figure 1H) and increased iHep cell formation (Figure 1I and Supplementary information, Figure S3B and S3C). KU55933, an ATM-specific inhibitor, was used to block ATM activation at the beginning of iHep induction. Phosphorylation of H2AX (γH2AX), a major downstream substrate of ATM^24^, was reduced after KU55933 treatment. In addition, KU55933 decreased p53 phosphorylation levels and facilitated formation of iHep cells (Supplementary information, Figure S3D and S3E). Intriguingly, in *ATM*^+/−^, KU55933-treated and *p53*^+/−^ iHep cells, hepatic genes were expressed at levels comparable to those in WT iHep cells (Supplementary information, Figures S3C, S3F and S1E). These results indicate that activation of the ATM-p53 pathway blocks hepatic conversion without interfering with hepatic gene induction.

We next asked whether hepatic conversion might induce extensive DNA damage, which would result in highly activated ATM^25^. DNA strand breaks were undetectable by the comet assay after 3TF transduction^26^ (Supplementary information, Figure S4A). In contrast, comet tails were readily detected after 1Gy γ-irradiation, which triggered p-ATM at a level lower than that in iHep induction (Supplementary information, Figure S4A). DNA strand breaks were also not detected by the TUNEL assay (Supplementary information, Figure S4B). RPA and 53BP1, key players in DNA damage repair^27^, were not activated after 3TF transduction as shown by immunofluorescence staining (Supplementary information, Figure S4C and S4D). Furthermore, genes involved in double-strand break repair, nucleotide excision repair and base excision repair^27^ were not upregulated after 3TF transduction (Supplementary information, Figure S4E). Nbs1 and ATMIN activate ATM in a DNA damage-dependent and -independent manner, respectively^28,29,30^. Interestingly, shRNA-mediated silencing of ATMIN, but not Nbs1, attenuated 3TF-induced ATM and p53 activation (Supplementary information, Figure S5A-S5D). Furthermore, ATMIN knockdown enhanced the generation of iHep cells (Supplementary information, Figure S5E), suggesting ATMIN-dependent phosphorylation of ATM. These findings indicate that it is unlikely that DNA damage, if any, causes highly phosphorylated levels of ATM during hepatic conversion.

### Early molecular events during hepatic lineage conversion

**Figure 2:**
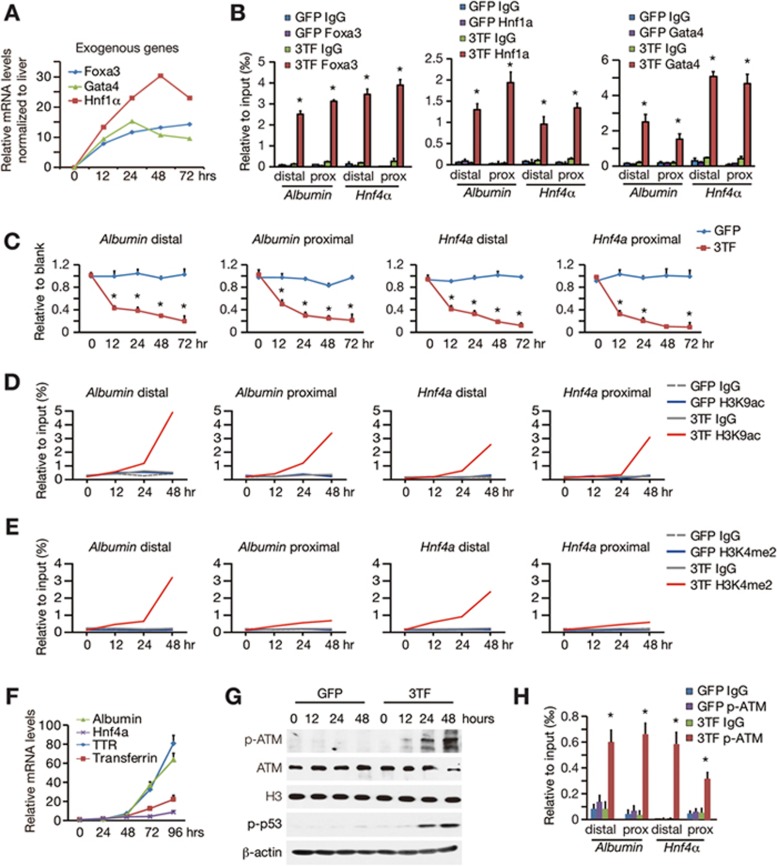
Early molecular events during hepatic conversion. **(A)** Expression level of 3TF was measured by qRT-PCR at different time points after transduction. **(B)** Binding of 3TF to regulatory regions of the *Albumin* and *Hnf4α* genes was analyzed by the ChIP assay. Distal and proximal binding sites were determined in Supplementary information, Figure S6. Data represent two independent experiments. **(C)** Chromatin opening at the *Albumin* and *Hnf4α* genes was measured by the micrococcal nuclease (MNase) digestion assay at different time points after 3TF transduction. Data represent two independent experiments. **(D**, **E)** Activated histone marks of H3K9ac **(D)** and H3K4me2 **(E)** on the *Albumin* and *Hnf4α* genes were examined by the ChIP-qPCR assay at different time points after 3TF transduction. **(F)** 3TF-induced hepatic gene expression was measured by qRT-PCR at different time points compared to TTFs. **(G)** p-ATM and p-p53 (Ser15) levels were analyzed by western blot analyses at different time points. **(H)** Binding of p-ATM to regulatory regions of the *Albumin* and *Hnf4α* genes was analyzed by the ChIP-qPCR assay at 48 h after 3TF transduction. Data represent two independent experiments. Error bars indicate SD. ^*^P< 0.05, Student's *t*-test.

To uncover the mechanism underlying ATM activation, we analyzed 3TF-induced molecular changes. We first characterized the regulation of the *Albumin* and *Hnf4α* genes, both of which are prominently induced by 3TF and are critical for the establishment of hepatic identity^12,13,31^. Twelve hours after transduction, 3TF were already expressed at levels comparable to those in liver cells (Figure 2A). Because Foxa3 and Gata4 act as pioneer factors to open closed chromatin during liver development^32,33^, we determined the chromatin opening after 3TF induction. Chromatin immunoprecipitation (ChIP) assays showed that 3TF bound upstream regulatory regions of the *Albumin* and *Hnf4α* transcriptional starting sites (Figure 2B and Supplementary information, Figure S6A). As a result of 3TF binding, the upstream regulatory regions of the *Albumin* and *Hnf4α* genes were opened at 12 h after 3TF transduction as determined by the micrococcal nuclease digestion assay (Figure 2C), suggesting that chromatin opening happens early during hepatic conversion. Marks of active genome regions, such as H3K9ac and H3K4me2, were also increased at the upstream regulatory regions of the *Albumin* and *Hnf4α* genes mainly at 48 h after 3TF transduction (Figure 2D and 2E and Supplementary information, Figure S6B). Finally, the mRNA levels of hepatic genes, including *Albumin* and *Hnf4α*, were induced at 72 h (Figure 2F). These data collectively revealed sequential molecular events after 3TF transduction.

Kinetic analyses of ATM and p53 phosphorylation showed that both ATM and p53 were activated as early as 24 h after 3TF transduction (Figure 2G and Supplementary information, Figure S7A-S7C). These time-course analyses indicated a temporal correlation between chromatin opening and ATM activation. Markedly, we found that p-ATM was recruited to open chromatin regions of the *Albumin* and *Hnf4α* genes after 3TF transduction (Figure 2H). Furthermore, 3TF-induced chromatin opening, H3K9 acetylation and p-ATM binding were validated on a panel of hepatic genes (Supplementary information, Figure S7D-S7F and Table S3). These results suggest that ATM activation is a molecular consequence associated with chromatin opening at a panel of hepatic gene loci.

### Baf60b is required for 3TF-induced ATM activation

**Figure 3:**
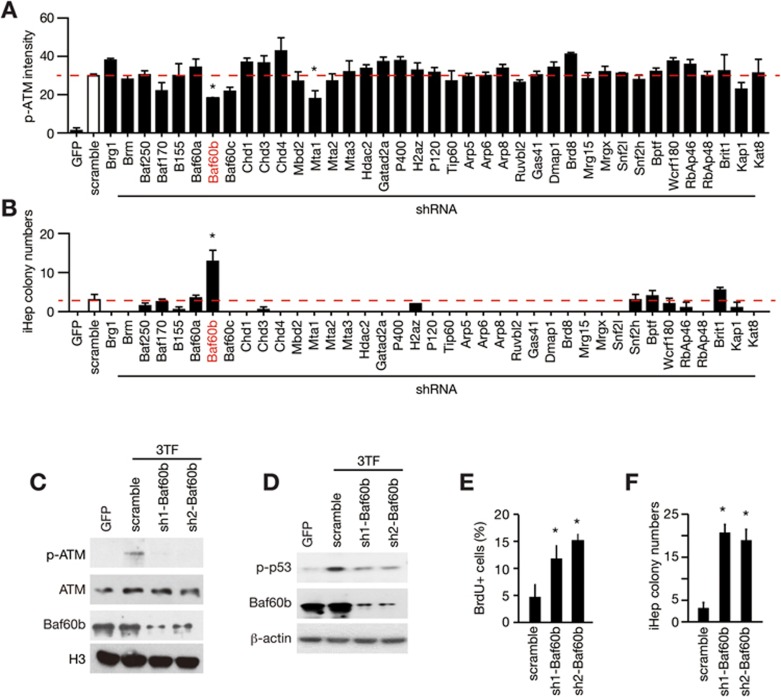
Baf60b mediates 3TF-induced ATM activation. **(A)** The hepatic conversion was induced in wild-type (WT) TTFs transfected with shRNAs against the indicated genes. Forty-eight hours after 3TF transduction, p-ATM levels were imaged by the Operetta system and quantified by Harmony software. *n* = 2 independent experiments, and 350-500 nuclei were analyzed for each shRNA in both experiments. **(B)** Numbers of iHep colonies were quantified 8 days after 3TF transduction. Data represent two independent experiments. **(C**-**F)** The role of Baf60b in ATM activation and iHep formation was confirmed by shRNAs targeting Baf60b CDS (sh1-Baf60b) and 3′UTR (sh2-Baf60b). **(C**, **D)** p-ATM and p-p53 (Ser15) levels were analyzed by the western blot assay. **(E)** Cell proliferation was determined by BrdU incorporation and staining. *n* = 5 fields for each group. **(F)** iHep colony numbers were quantified 8 days after 3TF transduction. *n* = 4 independent experiments for each group. Data represent two independent experiments. Error bars indicate SD. ^*^P< 0.05. Student's *t*-test.

We next characterized the connection between chromatin opening and ATM activation, and we sought to identify molecules mediating these two events. Chromatin remodeling complexes are the main regulatory complexes that open chromatin and allow DNA and histones to be accessible^34,35^. We silenced the expression of components of the SWI/SNF, CHD, INO80 and ISWI chromatin remodeling complexes by specific shRNAs (Supplementary information, Figure S8A). These shRNAs efficiently decreased the mRNA levels of target genes as determined by qRT-PCR (Supplementary information, Figure S8A). Knockdown of Baf60b and Mta1 reduced the activation of ATM after 3TF transduction (Figure 3A and Supplementary information, Figure S8B and S8C). Intriguingly, Mta1 knockdown significantly reduced cell number (Supplementary information, Figure S8D and S8E), suggesting that Mta1 silencing may have an adverse role in hepatic conversion. In contrast, depletion of Baf60b, a member of the SWI/SNF complex, markedly increased the generation of iHep colonies (Figure 3B). We confirmed the function of Baf60b by additional shRNAs that target Baf60b CDS (sh1-Baf60b) and 3UTR (sh2-Baf60b) (Supplementary information, Figure S8F). Both shRNAs significantly reduced p-ATM and p-p53 levels (Figure 3C and 3D and Supplementary information, Figure S8G). Baf60b knockdown alleviated proliferation arrest during hepatic conversion and eventually enhanced iHep formation (Figure 3E and 3F and Supplementary information, Figure S9A and S9B). Similar results were found using CRISPR/Cas9-mediated Baf60b-knockout cells (Supplementary information, Figure S9C-S9E). Notably, Bafb0b-silenced iHep cells showed comparable hepatic functions with control iHep cells, including albumin secretion, glycogen storage and drug metabolism (Supplementary information, Figure S9F and S9G), and they appeared to be non-tumorigenic (Supplementary information, Figure S9H). These findings demonstrate a regulatory role of Baf60b in ATM activation and iHep formation.

### Baf60b links chromatin opening to ATM activation

**Figure 4:**
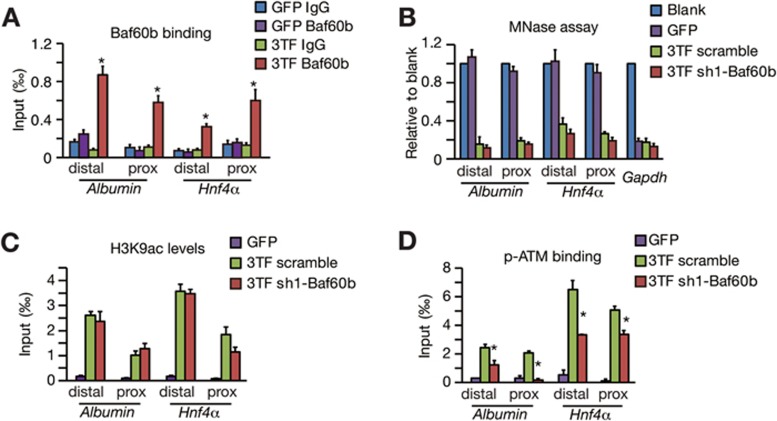
Baf60b links chromatin opening and ATM activation. **(A)** The binding of Baf60b at the regulatory regions of the *Albumin* and *Hnf4α* genes was determined by the ChIP-qPCR assay. **(B)** Chromatin opening at the *Albumin* and *Hnf4α* genes was measured by the micrococcal nuclease (MNase) assay in Baf60b-knockdown (sh1-Baf60b) TTFs and scramble controls after 3TF transduction. **(C)** H3K9ac on the *Albumin* and *Hnf4α* genes was examined in Baf60b-knockdown (sh1-Baf60b) TTFs and scramble controls by the ChIP-qPCR assay. **(D)** The binding of p-ATM to the *Albumin* and *Hnf4α* genes was analyzed by the ChIP-qPCR assay. sh1-Baf60b-mediated Baf60b silencing attenuated the p-ATM binding. Data represent two independent experiments. Error bars indicate SD. ^*^P< 0.05. Student's *t*-test.

We next analyzed whether Baf60b mediates chromatin opening and ATM activation. To that end, we first determined whether Baf60b is in the SWI/SNF complex. Immunoprecipitation assays showed that HA-tagged Baf60b interacted with the SWI/SNF complex members, including Brg1, a core ATPase of the SWI/SNF complex (Supplementary information, Figure S10A); but Baf60a and Baf60c were excluded from the complex containing Baf60b (Supplementary information, Figure S10B). Mass spectrometry analysis supported these findings, indicating that the complex is stable during hepatic conversion (Supplementary information, Figure S10C and Table S10). ChIP assays also showed that Baf60b, Brg1 and Baf170 bound to the open chromatin regions of a panel of hepatic gene loci (Figure 4A and Supplementary information, Figure S11A-S11C and Tables S3-S4). Interestingly, Brg1 silencing caused reduced chromatin opening and H3K9ac level in hepatic genes (Supplementary information, Figure S12A-S12B and Table S5), whereas Baf60b depletion did not affect these changes (Figure 4B, 4C and Supplementary information, Figure S12C, S12D and Table S5). Given that the levels of Brg1-bound Baf60a and Baf60c were increased in Baf60b-deficient cells (Supplementary information, Figure S13A) and silencing all Baf60 members led to impaired chromatin opening at a panel of hepatic gene loci (Supplementary information, Figure S13B and Table S6), it is likely that Baf60a or Baf60c replaced Baf60b functions in Baf60b-deficient cells. Together, these results show that Baf60b is in the SWI/SNF complex and is located at open chromatin regions. A kinetic analysis of the Baf60b and p-ATM binding revealed that Baf60b and p-ATM were sequentially recruited to open chromatin regions of hepatic genes (Supplementary information, Figure S14A and Table S7), suggesting that chromatin recruitment of these two proteins might be associated. Importantly, we found that Baf60b knockdown significantly reduced the binding of p-ATM to open chromatin regions of hepatic genes (Figure 4D and Supplementary information, Figure S14B and Table S8). These data indicate that Baf60b is in the SWI/SNF complex and mediates ATM recruitment to the open chromatin regions at a panel of hepatic gene loci.

### Baf60b facilitates the recruitment of ATM to the SWI/SNF complex

**Figure 5:**
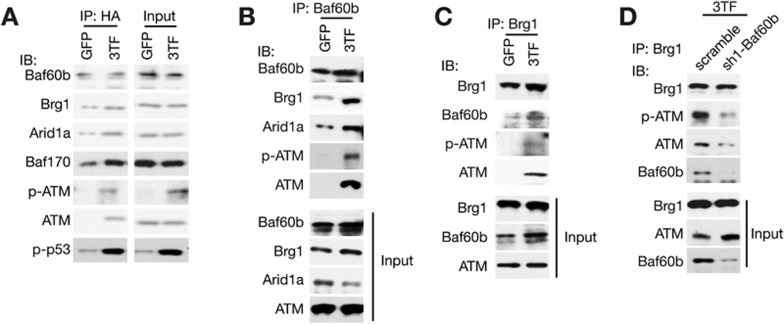
Baf60b facilitates ATM recruitment to the SWI/SNF complex and ATM activation. **(A)** Analyses of the interaction among Baf60b, the SWI/SNF complex, ATM, p-ATM and p-p53. The hepatic conversion was induced in TTFs transduced with HA-tagged Baf60b. Cell lysates were immunoprecipitated with an HA antibody or control IgG followed by immunoblot (IB) assays with antibodies against the indicated proteins. **(B**, **C)** 3TF-transduced TTF lysates were immunoprecipitated with antibodies against endogenous Baf60b **(B)** or against endogenous Brg1 **(C)** followed by IB analyses with antibodies against indicated proteins. **(D)** Baf60b was silenced by sh1-Baf60b in TTFs. Forty-eight hours after 3TF transduction, lysates of TTFs were immunoprecipitated with Brg1 antibody followed by IB analysis with ATM and p-ATM antibodies.

Because the SWI/SNF complex contains no kinase^34,35^ and chromatin localization of ATM has an important role in its activation^25^, we characterized whether Baf60b mediates ATM recruitment to open chromatin through the SWI/SNF complex and therefore facilitates its activation. Baf60b was in the SWI/SNF complex as shown by immunoprecipitation assay during hepatic conversion (Figure 5A). Notably, 3TF transduction triggered an interaction between Baf60b and ATM (Figure 5A). We further confirmed the interaction of endogenous Baf60b with ATM and the SWI/SNF complex using monoclonal antibodies specific to Baf60b (Figure 5B). In addition, reverse co-immunoprecipitation using antibodies against Brg1 confirmed that 3TF transduction triggered a remarkable interaction between Brg1 and ATM (Figure 5C). Moreover, Baf60b knockdown depleted interactions between Brg1 and ATM (Figure 5D). The 3TF-triggered interaction of Baf60b with the SWI/SNF complex and ATM was also validated by mass spectrometry (Supplementary information, Figure S10C and Table S10). In line with ATM recruitment to the SWI/SNF complex, p-ATM was detected in the Baf60b-precipitated complex (Figure 5A-5C). Furthermore, reduced interaction between ATM and Brg1 was correlated with decreased p-ATM in Baf60b-knockdown cells (Figure 5D). In addition, p53 and phosphorylated p53, as a substrate of p-ATM, were detected in the Baf60b-precipitated complex (Figure 5A and Supplementary information, Figure S10C and Table S10), suggesting that ATM recruitment and activation are associated. Given that ATMIN controls ATM activation during hepatic conversion (Supplementary information, Figure S5), we analyzed ATM binding in ATMIN-knockdown cells to further dissect ATM recruitment and activation. ATMIN knockdown decreased the amount of Baf60b-bound p-ATM (Supplementary information, Figure S15A) and the recruitment of p-ATM to open chromatin regions of hepatic genes (Supplementary information, Figure S15B and Table S9). In contrast, the amount of Baf60b-bound ATM was unchanged in ATMIN-knockdown cells (Supplementary information, Figure S15A). These results demonstrate a role of Baf60b in recruiting ATM to the SWI/SNF complex after 3TF transduction.

### The nuclear location sequence and SWIB domains are necessary for Baf60b-mediated ATM activation

**Figure 6:**
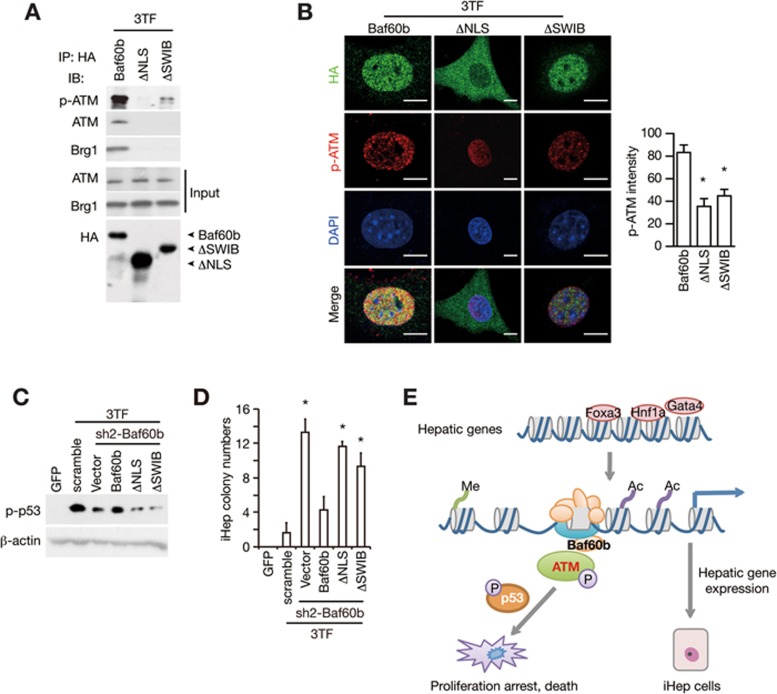
The NLS and SWIB domains are necessary for Baf60b-dependent ATM activation. **(A)** HA-tagged Baf60b and two Baf60b mutants (ΔNLS and ΔSWIB) were individually transduced into TTFs. Interactions among Baf60b mutants, Brg1, ATM and p-ATM were determined. **(B**-**D)** ΔNLS, ΔSWIB and HA-tagged Baf60b were transduced into Baf60b-silenced TTFs in which the endogenous 3′UTR of Baf60b mRNA was targeted by sh2-Baf60b. 3TF-induced p-ATM was analyzed by immunofluorescence staining and quantified by LAS AF Lite; *n* = 6 cells were analyzed for each group **(B)**. p-p53 (Ser15) protein level was determined by the western blot assay **(C)** and iHep colony numbers were quantified 8 days after iHep induction; *n* = 4 independent experiments **(D)**. **(E)** A schematic model showing that a repressive mechanism involving Baf60b-mediated ATM activation senses chromatin opening and limits hepatic conversion. Error bars indicate SD. ^*^P< 0.05. Student's *t*-test.

We next investigated whether Baf60b-mediated ATM recruitment is required for ATM activation. We cloned two Baf60b mutants containing the deletion of either the nuclear location sequence (ΔNLS) or the conserved SWIB domain (ΔSWIB) of Baf60b (Supplementary information, Figure S16A). The full-length HA-tagged Baf60b was used as a control. Immunofluorescence staining confirmed that ΔNLS localized in the cytoplasm, whereas the ΔSWIB mutant and the HA-tagged Baf60b control localized in the nucleus (Supplementary information, Figure S16B). Due to the cytoplasmic location, the ΔNLS mutant showed no interaction with the SWI/SNF complex (Supplementary information, Figure S16C) and was unable to recruit ATM after 3TF transduction (Figure 6A). The ΔSWIB mutant localized in the nucleus, but it lost the capability to interact with the SWI/SNF complex (Supplementary information, Figures S10C and S16C) and to recruit ATM as shown by immunoprecipitation and mass spectrometry (Figure 6A and Supplementary information, Figure S10C and Table S10). To determine their role in ATM activation, these mutants were expressed in Baf60b-silenced cells in which the endogenous 3′UTR of Baf60b was targeted by sh2-Baf60b. It was notable that these two mutants caused remarkably reduced p-ATM levels compared to the full-length Baf60b control (Figure 6B). As a consequence, both ΔNLS and ΔSWIB mutants decreased p53 phosphorylation level (Figure 6C) and improved the formation of hepatic colonies (Figure 6D). These results showed that ATM recruitment to the SWI/SNF complex facilitates its full activation during lineage conversion.

## Discussion

In this study, we identified a molecular basis for the barrier against lineage conversion using 3TF-induced iHep cells as an experimental system. In line with studies on other lineage conversions^36,37,38,39^, the binding of 3TF to the compact chromatin of lineage-specific genes initiates the hepatic lineage conversion. Transduction of 3TF triggers the chromatin remodeling complex to open chromatin of hepatocyte-specific genes and to facilitate their expression (Figure 6E). Importantly, we found that 3TF induce chromatin opening and activate, in parallel, the ATM-p53 pathway, which acts as a negative regulatory loop halting hepatic conversion (Figure 6E). Mechanistically, Baf60b, a component of the SWI/SNF complex, binds to open chromatin regions of hepatic genes and facilitates ATMIN-dependent ATM activation by recruiting ATM to the SWI/SNF complex. Together, we show that ATM activation blocks hepatic conversion by sensing chromatin opening at a panel of hepatic gene loci (Figure 6E), suggesting that an inappropriately high level of newly opened chromatin regions impedes cell identity conversion by triggering the activation of a “chromatin remodeling checkpoint”. Our findings suggest a paradigm of how a differentiated cell resists dramatic cell identity changes.

It is remarkable that the ATM-p53 pathway limits lineage conversion in three seemingly unrelated scenarios, i.e., oncogenic transformation, iPS cell generation and hepatic conversion^17,18,19,20,21,40^. It remains to be identified whether it is simply a coincidence or whether there are common fundamental mechanisms restricting the plasticity of cell identity. Given that both oncogenic transformation and iPS cell generation are associated with epigenetic remodeling^41,42,43^, it would be worth testing whether in these two processes the ATM-p53 pathway senses chromatin opening and blocks cell identity conversion. Indeed, our preliminary data supported that BAf60b may also be involved in ATM activation during iPS cell induction, although to a lower degree (Supplementary information, Figure S17 and Table S11). It is possible that chromatin remodelers play lineage-specific roles in various cell identity conversion systems.

As a sensor of DNA damage, ATM is activated in response to DNA double-strand break. Activated ATM transduces the signal to DNA damage repair proteins and promotes p53 phosphorylation and stability to maintain the DNA integrity and prevent tumorigenesis^44^. Interestingly, ATM is also activated by chloroquine, hypotonic stress and chromatin-modifying drugs without DNA damage^45^, suggesting that ATM activation could result from changes in chromatin conformation. Transcription factor-induced cell lineage conversion is a specific process during which large-scale chromatin remodeling occurs. Our results confirmed that ATM was activated and recruited to 3TF-induced chromatin opening at a panel of hepatic genes. It is interesting to postulate that ATM might be a guard protein that senses the changes in chromatin conformation and helps to maintain the specific identity of a given cell.

We found that Baf60b, a member of the SWI/SNF chromatin remodeling complex, was the key for ATM to sense the chromatin opening. Interestingly, Baf60b appeared not to be essential for chromatin opening. One possible explanation is that after Baf60b depletion, Baf60a and Baf60c replaced Baf60b function in opening target chromatin. However, Baf60a and Baf60c could not replace Baf60b in ATM recruitment. shRNA screening data showed that there are low or no compensatory functions between Baf60a/b/c in iHep reprogramming. Specifically, Baf60a knockdown displayed no apparent effect on iHep formation, whereas Baf60c knockdown blocked iHep reprogramming (Figure 3B), suggesting limited ability of Baf60 subunits to compensate for each other in iHep formation. These data also suggested a possible role of Baf60c in mediating chromatin opening. There might be a switch between Baf60 members during chromatin opening and ATM activation. Nevertheless, given that iHep formation is a long-time procedure, the contribution of individual Baf60 members at late stage of iHep induction needs further characterization.

Our results also partially explained why *in vitro* hepatic conversion is a low-efficiency process. By silencing Baf60b or inactivating the ATM-p53 pathway, the hepatic conversion was allowed to occur at significantly high efficiency. Specifically, Baf60b inactivation would be superior to the inhibition of the p53 pathway by keeping the DNA damage response intact. A careful investigation of the response to epigenetic remodeling might also improve efficiency and quality of other lineage conversions.

## Materials and Methods

### Mice and isolation of TTFs

All mice used in this study were maintained under specific pathogen-free conditions at the animal facility of Institute of Biochemistry and Cell Biology, Chinese Academy of Sciences. TTFs were isolated from 4-week-old male C57/B6 mice or genetically modified mice. ∼2 cm length of tail tip was cut and washed with 70% ethanol and PBS. After dermis was peeled away, the remaining part was placed into a 60-mm collagen-I-coated dish in 5 ml DMEM (Hyclone) containing 10% FBS (Gibco). After 5 days incubation, fibroblasts that migrated out were trypsinized and expanded to a 10-cm collagen-I-coated dish. We used TTFs at passage 3 for iHep cell induction. All mouse experiments were approved by the Institutional Animal Care and Use Committee (IACUC) of the Institute of Biochemistry and Cell Biology and performed in accordance with the committee's guidelines.

### iHep cell induction

iHep cells were generated as previously described^12^. Briefly, 10^5^ TTFs at passage 3 were seeded on a collagen-I-coated 6 cm dish and transfected with lentiviruses carrying 3TF. Cells were collected for further analyzing at different time points according to specific experiment. We quantified the colonies at day 8 or later after iHep induction. At this time point, epithelial cell colonies were visible and could be quantified. In previous study, we have demonstrated that these individual iHep colonies were similar to each other^12^. Some of the colonies were additionally confirmed as iHep cells by immunofluorescence staining for Albumin and E-cadherin.

### Molecular cloning and lentivirus production

The constructions of 3TF were described previously^12^. Baf60b, ΔNLS and ΔSWIB cDNA with HA-Tag at N-terminus were cloned into the modified pWPI plasmid (Addgene). The NLS domain distributes from 122 aa to 207 aa of Baf60b protein as indicated by our results. The SWIB domain (307 aa to 385 aa) was determined according to the previous report^46^. For shRNA-mediated gene silencing, DNA oligonucleotides targeting each gene were inserted into the *Age*I and *EcoR*I restriction enzyme sites of the pLKO.1 plasmid, respectively. DNA sequences were mainly obtained from Sigma MISSION shRNA library. The oligonucleotide sequences are provided in Supplementary information, Table S12. Constructed pWPI or pLKO.1 plasmids were introduced into 293FT cells together with packaging plasmid psPAX2 (Addgene) and envelope plasmid pMD2.G (Addgene). After 48 h of incubation, lentiviruses were collected and stored at −80 °C.

### Immunofluorescence staining assays

Cells were fixed with 4% paraformaldehyde for 15 min at room temperature, and then blocked with PBS containing 0.25% Triton X-100 (Sigma) and 3% BSA (Sigma) for 1 h at room temperature. The cells were then incubated with primary antibodies at 4 °C overnight, washed with PBS, and incubated with appropriate fluorescence-conjugated secondary antibody for 1 h at room temperature in the dark. Nuclei were stained with DAPI (Sigma). Primary and secondary antibodies were diluted in PBS containing 3% BSA. Images were captured at room temperature using an Olympus microscope (IX71) or a confocal microscope (TCS SP5; Leica) with a 63× oil objective. The p-ATM intensity was quantified by software LAS AF Lite (Leica). Antibodies used for immunofluorescence are as follows: rabbit anti-E-cadherin (Cell Signaling, 1:200), mouse anti-albumin (R&D, 1:200), mouse anti-p-ATM (Ser1981) (Thermo, 1:200), rabbit anti-HA-Tag (Cell Signaling, 1:200), rat anti-RPA32 (Cell Signaling, 1:200), rabbit anti-53BP1 (Novus, 1:200), goat anti-Nanog (R&D, 1:100), mouse anti-SSEA1 (R&D, 1:100), Cy3-conjugated donkey anti-mouse/rabbit/rat/goat IgG (Jackson Laboratories, 1:1 000), Cy5-conjugated goat anti-rabbit/mouse IgG (Jackson Laboratories, 1:1 000).

### 5-bromodeoxyuridine (BrdU) incorporation assay

Cells were incubated with BrdU (BrdU Labeling Reagent, Ready-to-use, Invitrogen, 1:100 diluted in culture medium) for 6 h at 37 °C. Cells were washed with PBS and fixed in acid ethanol (90% ethanol, 5% acetic acid, 5% H_2_O) for 30 min at room temperature, followed by incubation in 2 M HCl for 20 min at room temperature. 0.1 M sodium borate (pH 8.5) was added to the cells and incubated for another 2 min. Cells were washed with PBS and incubated with BrdU antibody (Cell Signaling, 1:1 400) in 3% BSA-PBS at 4 °C overnight. After washed with PBS, the cells were incubated with Cy3-conjugated donkey anti-mouse IgG. Nuclei were stained with DAPI (Sigma). The BrdU-positive cells were counted using an Olympus microscope (IX71).

### SA-β-Gal staining

SA-β-Gal staining was performed according to the manufacturer's instruction (Beyotime). Briefly, 5 × 10^4^ TTF cells were placed in a 6-well plate and transduced with 3TF or empty vector and cultured for 6 days. After being washed with PBS, cells were fixed and then incubated with staining working solution overnight at 37 °C. The senescent cells were counted under an Olympus microscope (IX71).

### Flow cytometry

For apoptosis analysis, cells were collected at day 6 after 3TF or empty vector transfection. Apoptotic cells were detected by Annexin V Apoptosis Detection Kit APC (eBioscience) and analyzed by FACS Calibur. The production of reactive oxygen species (ROS) was detected 48 h after 3TF transduction using CellROX^TM^ Deep Red Reagent (Invitrogen). Cells were incubated with CellROX^TM^ Deep Red Reagent at a final concentration of 5 μM for 30 min at 37 °C. The labeled cells were washed with PBS and analyzed by FACS Calibur (Becton Dickinson).

### Comet assay

The microscope slide was pre-treated with 10% polylysine and coated with a thin 1% normal melting point agarose (NMA) gel. Cells were collected at 48 h after 3TF transfection, suspended in 1% low melting point agarose (LMA) and embedded within the NMA gel on the slide. The slide was put in the lysis buffer (2.5 mM NaCl, 100 mM EDTA, 10 mM Tris base, 1% Triton X-100) at 4 °C for 1 h to remove the cellular proteins. The DNA was allowed to unwind in the alkaline pH conditions (300 mM NaOH, 1 mM EDTA) at room temperature for 1.5 h and then electrophoresed at 25 V, 300 mA for 20 min. The DNA was stained with ethidium bromide. Pictures were taken by an Olympus microscope (BX51). The comet tail length was measured by CASP software.

### Micrococcal nuclease (MNase) digestion assay

The MNase digestion assay was performed as described before with some modifications^47^. 5 × 10^5^ cells were collected and suspended in Buffer A (10 mM HEPES pH7.9, 1.5 mM MgCl_2_, 10 mM KCl, 0.5 mM DTT) supplemented with protease inhibitor cocktail (Sigma) on ice for 15 min. NP40 was then added to 0.5% concentration followed by vortex for 20 s. The nuclei pellets were collected by centrifuged at 4 000 rpm for 5 min. The nuclei was washed with 2.5 ml of MNase digestion buffer (10 mM Tris-HCl pH7.4, 15 mM NaCl, 60 mM KCl, 0.15 mM spermine, 0.5 mM spermidine) and centrifuged to collect the pellet. The nuclei was resuspended in 1 ml of MNase digestion buffer containing 1 mM CaCl_2._ 100 μl of nuclei were transferred to a series of microcentrifuge tubes containing 2 U of MNase (Takara). Samples were incubated at room temperature for 5 min. 80 μl of MNase digestion buffer and 20 μl MNase stop buffer (100 mM EDTA, 10 mM EGTA pH7.5) were added to each sample. Proteinase K was added to a final concentration of 0.2 mg/ml. 10 μl of 20% SDS was added. After incubation at 55 °C for 4 h, DNA was purified using MinElute PCR Purification Kit (Qiagen) and characterized by qPCR. Primer sequences are provided in Supplementary information, Tables S1 and S13.

### Chromatin immunoprecipitation

Cells were washed with PBS and crosslinked with 1% formaldehyde for 10 min. A final concentration of 0.2M Glycine was added to quench the crosslinking for 5 min, and then the cells was washed with PBS. Nuclei were isolated and resuspended in lysis buffer (10mM Tris-HCl, pH 8.0, 200mM NaCl, 1mM EDTA, 0.5mM EGTA, 0.1% Na-Deoxycholate, 0.5% N-lauroylsarcosine sodium salt, 0.2% SDS, protease inhibitor) for sonication, followed by sonication (Covaris S220) to yield DNA fragments around 200-500 bp. The lysate was centrifuged. A certain amount of supernatant was used for input. After adding the incubation buffer to a final concentration of 1× RIPA (1% Triton X-100, 0.1% SDS, 0.1% Na-Deoxycholate, in TE buffer) with 0.4 M NaCl and protease inhibitor, DNA was co-immunoprecipitated using ChIP-grade antibodies against Foxa3, Gata4 and Hnf1a (Santa Cruz), H3K9ac and H3K4me2 ChIP-grade antibodies (Abcam), p-ATM (Ser1981) antibody (Thermo), and Baf60b antibody (Abnova) and Brg1(Santa Cruz) and Baf170 (Bethyl laboratories) or control IgG (normal mouse and rabbit IgG from Millippore, normal goat IgG from Santa Cruz) at 4 °C overnight. The immunoprecipitates were collected by incubating with Dynabeads Protein G (Life Technologies) at 4 °C for 2 h, and sequentially washed with RIPA buffer (1% Triton X-100, 0.1% SDS, 0.1% Na- Deoxycholate, in TE) twice, RIPA buffer with 0.3 M NaCl twice, LiCl buffer (0.25 M LiCl, 0.5% NP40, 1% deoxycholic acid, 1 mM EDTA, 10 mM Tris-HCl pH 8.0) twice, TE buffer (10 mM Tris-HCl pH 8.0, 1 mM EDTA) with 0.2% Triton X-100 once and TE buffer once. The immunoprecipitates and the input DNA were reverse-crosslinked with 0.2 mg/ml proteinase K at 65 °C for 2 h and finally eluted from the beads using Elution buffer (Qiagen) and treated with RNase A at 37 °C for 30 min. DNA fragments and input DNA were purified using MinElute PCR Purification Kit (Qiagen) and the DNA concentration is quantified by Qbit (Life Technologies). The same amount of DNA in each panel of experiment is used for qPCR. Primer sequences are provided in Supplementary information, Tables S1 and S13.

To characterize a panel of hepatic genes in this study using ChIP-qPCR, we tested 34 candidate hepatic genes based on published data^48,49,50^. Next, we designed PCR primer pairs for 205 sites located in the 34 genes to identify 3TF-binding loci (Supplementary information, Table S1). By ChIP-qPCR assay, we found 81 sites with enriched 3TF-binding in 30 out of the 34 candidate genes (Supplementary information, Table S2). We selected 32 sites on 24 genes for further analyses (Supplementary information, Table S3). The 32 sites were selected by their distant location from each other.

### Immunoprecipitation and immunoblot analysis

For immunoprecipitation assay, TTFs were transfected with 3TF and respective cDNA clone (HA-Baf60b, HA-ΔNLS, HA-ΔSWIB) in pWPI vector (modified from Addgene). Cells were suspended in Buffer A (10 mM HEPES pH7.9, 1.5 mM MgCl_2_, 10 mM KCl, 0.5 mM DTT) supplemented with protease inhibitor cocktail (Sigma) on ice for 15 min. NP40 was then added into a final concentration of 0.5% followed by vortex for 20 s. The nuclear pellets were collected by centrifugation at 4 000 rpm for 5 min. The nuclei were lysed in buffer (50 mM Tris-HCl pH 7.4, 150 mM NaCl, 1 mM EDTA, 1% Triton X-100 and 0.1% NP40) supplemented with protease inhibitor cocktail. Lysates were incubated with appropriate antibody or IgG at 4 °C overnight before adding Protein G-conjugated Dynabeads for 2 h. The beads were washed three times with lysis buffer and eluted with SDS loading buffer by boiling for 5 min, and subjected to immunoblot analysis. For immunoblot analysis, the samples were separated by SDS-PAGE, and transferred to a PVDF membrane (Millipore). The membrane was blocked with 5% (w/v) reagent-grade nonfat milk (Cell Signaling Technology) and incubated with primary antibodies at 4 °C overnight followed by secondary antibody incubation. The protein bands were visualized using Clarity^TM^ Western ECL substrate (Bio-Rad).

### Mass spectrometry and data analysis

The Co-IP samples were separated on an 8% SDS-PAGE, and then stained with Coomassie Blue. The gel lanes were cut into eight bands per sample and in-gel trypsin digestion was performed strictly following a reported protocol^50^. Liquid chromatography separation was performed on a Proxeon Easy-nanoLC 1000 system (ThermoFisher Scientific). The LC column (15-cm length, 75-μm inner diameter) was packed in-house with ReproSil-Pur C18-AQ 3 μm resin (Dr Maisch GmbH) in 100% methanol. For high mass accuracy data acquisition, the Orbitrap Fusion Tribrid mass spectrometer (ThermoFisher Scientific) was used and equipped with a nano-electrospray ion source (Proxeon Biosystems, now ThermoFisher Scientific). The peptides were dissolved in 0.1% FA, loaded onto the column and separated with a linear gradient of 4%-30% buffer B (ACN with 0.1% formic acid) at a flow rate of 300 nL/min over 43 min. The Fusion was operated using 'high-low' mode, in which during a maximum 3 s cycle time, the most abundant multiply charged parent ions were selected with high resolution (120 000, 200 m/z) from the full scan (300-1500 m/z) for HCD fragmentation. For MS/MS spectra, ion trap detector was used by a “rapid” scan rate with a target value of 2e4 (AGC control enabled) and an isolation window of 2 m/z. The normalized collision energy was set to 33%.

Raw data files were processed and analyzed using the MaxQuant software (Version. 1.5.2.8). The MS/MS spectra were searched against the mouse Uniprot protein database (201405) using the Andromeda search engine. The spectra were searched with a mass tolerance of 6 ppm in MS mode, 0.5 Da in HCD MS/MS mode, strict trypsin specificity, and allowing up to 2 missed cleavage sites. The minimum required peptide length was 6 amino acids. Cysteine carbamidomethylation was set as a fixed modification, whereas N-acetyl protein, oxidized methionine and acetylation of lysine were searched as variable modifications. The peptide and protein false discovery rate (FDR) was fixed at no more than 0.01. The LFQ intensity was used to quantify the abundance of proteins identified in each sample after normalization by total protein intensity and the intensity of Baf60b (Smarcd2).

### shRNA screening of chromatin remodelers for ATM activation

TTFs were transduced with lentivirus carrying shRNA targeting specific chromatin remodelers, and re-seeded to 24-well plate at the density of 15 000 cells per well followed by transfection with 3TF lentiviruses. p-ATM activation was analyzed by immunofluorescence quantification 48 h after 3TF transfection. Plate scan and image collection were performed on the Operetta HTS imaging system (PerkinElmer) at 20× magnification with 15 fields of view, Images were then analyzed with Harmony (PerkinElmer). iHep cell colony numbers were counted at day 8 after 3TF transduction.

### Glycogen storage, DiI-ac-LDL uptake, CYP metabolism assay, albumin ELISA and tumor generation assays

WT TTFs were infected with lenti-virus carrying shNT, sh1-Baf60b respectively for 48 h. Cells were transduced with 3TF for iHep cell generation. The hepatic function assays were done at day 21 after 3TF transfection. Glycogen storage was quantified by colorimetric measurement by Glycogen Assay Kit (Abnova). iHep cells were stained by DiI-ac-LDL (Invitrogen) following the manufacturer's instructions. For the measurement of CYP enzyme induction, iHep cells were cultured in Block's medium supplemented with 3-methylcholanthrene (25 μM) and sodium phenobarbital (1 mM) for 48 h. Cells were incubated with substrate in 200 μl incubation medium at 100 μM for 3 h at 37 °C. To stop the reaction, 800 μl of cold methanol was added and centrifuged. The supernatants were collected for measurement of metabolized compounds by LC-MS/MS (Agilent 1200 HPLC and ABI 4000 mass-spectrometer). Total cell protein amount was used to normalize the data. The amount of secreted Albumin was determined by the mouse Albumin ELISA kit (Bethyl Laboratory) according to the manufacturer's instructions.

For the tumor generation assay, human hepatoma cell line SNU398 was used as positive control. Three nude mice were injected with 3 × 10^6^ iHep cells in the left subcutaneous flank and 3 × 10^6^ SNU398 cells in the right subcutaneous flank. Tumor formation was examined 8 weeks after injection.

### iPS cell generation and characterization

WT TTFs were infected with lentiviruses carrying shNT, sh1-Baf60b and sh2-Baf60b respectively for 48 hours. Cells were reseeded and transfected with retrovirus carrying Oct4, Sox2, Klf4, and c-Myc for iPS cell induction^10^. The iPS cells were maintained on mitomycin C-treated MEF in DMEM/F12 culture medium supplemented with 20% KnockOut serum replacement, 0.1 mM non-essential amino acids, 1 mM L-glutamine, 0.1 mM β-mercaptoethanol and LIF. iPS colonies were picked for expansion around 14 days after transduction. iPS cells were characterized for alkaline phosphatase activity following the procedures in Alkaline Phosphatase kits (Sigma-Aldrich). AP positive colonies were counted to quantify the iPS induction efficiency at day 14 after transduction. The induction of Nanog and SSEA1 was measured by immunofluorescence staining. Pluripotent genes induction was determined by qRT-PCR. Primer sequences are provided in Supplementary information, Table S14.

### CRISPR/Cas9-mediated Baf60b knockout

We purchased *Baf60b*^+/−^ (Smarcd2^tm1.1 (KOMP)Vlcg^) mice from Knockout Mouse Project (KOMP) Repository located at University of California Davis. We were not able to obtain *Baf60b*^−/−^ mice by breeding Baf60b^+/−^ mice. Baf60b-specific sgRNA oligos were designed by the CRISPR website (http://crispr.mit.edu/) targeting sequences at exon 4 (5′-CCTCTCGAAAGCTAAAAGATCC-3′). The oligos were cloned into the lentiCRISPR V2 plasmid^52^. For efficient Baf60b knockout, we used *Baf60b*^+/−^ TTF cells as starting cells with serial lentiCRISPR V2-sgBaf60b virus transfection. We used puromycin to select the transfected cells. We used mixed Baf60b-knockout cells in the experiments. Genomic DNA was extracted from the mixed cells and the Baf60b knockout efficiency was analyzed by amplicon sequencing. Out of the 15 sequenced *Baf60b* genome PCR fragments, 14 showed nonsense mutations in the targeted region of the *Baf60b* gene (data not shown).

### Statistics

All data are presented as the mean + SD. For statistic evaluation, unpaired Student's *t*-test was applied for calculating statistical probability in this study. The differences were considered significant when one-tailed *P*< 0.05.

## Author Contributions

SJ and LH conceived the project. SJ performed most experiments with the support from LZ. LZ constructed the shRNA lentiviral vectors and performed the ChIP-PCR assay with the help from Yawei G and SG. Yimeng G conducted iPS reprogramming and characterization. YY designed the sgRNA for Baf60b. XZ analyzed the ChIP-seq data. SJ and RL conducted mass spectrum and data processing under the conduction of RZ and LL. JC was responsible for the mouse maintenance. CH and GW were involved in experimental design, data analyses and manuscript writing. SJ and LH wrote the manuscript.

## Competing Financial Interests

The authors declare no competing financial interests.

## Supplementary Information

Supplementary information, Figure S1 Inhibition of p53 enhances iHep formation.

Supplementary information, Figure S2 Unchanged levels of oncogenes and ROS during 3TF-induced hepatic conversion.

Supplementary information, Figure S3 ATM inactivation facilitates iHep formation.

Supplementary information, Figure S4 DNA damage is not detected in hepatic conversion.

Supplementary information, Figure S5 ATMIN controls 3TF-induced ATM activation.

Supplementary information, Figure S6 Analyses of upstream regulatory regions of *Albumin* and *Hnf4α* genes.

Supplementary information, Figure S7 Time-course analyses of ATM and p53 activation during hepatic conversion.

Supplementary information, Figure S8 Validation of Baf60b in controlling ATM activation.

Supplementary information, Figure S9 Baf60b inhibition facilitates iHep formation.

Supplementary information, Figure S10 Baf60b recruited ATM and p53 to the SWI/SNF complex.

Supplementary information, Figure S11 Analyses of the binding of the SWI/SNF complex to hepatic gene loci.

Supplementary information, Figure S12 Chromatin opening by Brg1 and Baf60b.

Supplementary information, Figure S13 Baf60a and Baf60c replace the chromatin-remodeling function of Baf60b in Baf60b-deficient cells.

Supplementary information, Figure S14 Baf60b mediates ATM recruitment.

Supplementary information, Figure S15 ATMIN is responsible for phosphorylation of Baf60b-recruited ATM.

Supplementary information, Figure S16 Baf60b-mediated ATM recruitment facilitates ATM activation.

Supplementary information, Figure S17 Baf60b depletion facilitates iPS cell formation.

Supplementary information, Table S1 3TF-binding candidate sites

Supplementary information, Table S2 3TF-binding at candidate sites as determined by the ChIP assay

Supplementary information, Table S3 Chromatin opening, p-ATM binding and Baf60b binding to hepatic gene sites

Supplementary information, Table S4 Brg1 and Baf170 binding in hepatic genes

Supplementary information, Table S5 Chromatin remodeling complex controls chromatin opening and active histone modification

Supplementary information, Table S6 Chromatin opening in Baf60a/b/c triple knockdown cells

Supplementary information, Table S7 Baf60b and p-ATM binding at 12 and 24 hours after induction of hepatic conversion

Supplementary information, Table S8 p-ATM binding in Baf60b silenced cells

Supplementary information, Table S9 p-ATM binding in ATMIN silenced cells

Supplementary information, Table S10 Mass spectrometry analyses of Baf60b-binding proteins

Supplementary information, Table S11 Baf60b and p-ATM binding at 48 hours after induction iPS cells

Supplementary information, Table S12 shRNA sequences

Supplementary information, Table S13 ChIP PCR primers

Supplementary information, Table S14 qPCR primers
