# Supplementary material for: Baf60b-mediated ATM-p53 activation blocks cell identity conversion by sensing chromatin opening
Source: Cell Res. 2017 Mar 17;27(5):642–56. doi: 10.1038/cr.2017.36 (PMC5520852; doi:10.1038/cr.2017.36)
Supplement: Supplementary information, Figure S1 — Inhibition of p53 enhances iHep formation. [file cr201736x1.pdf]

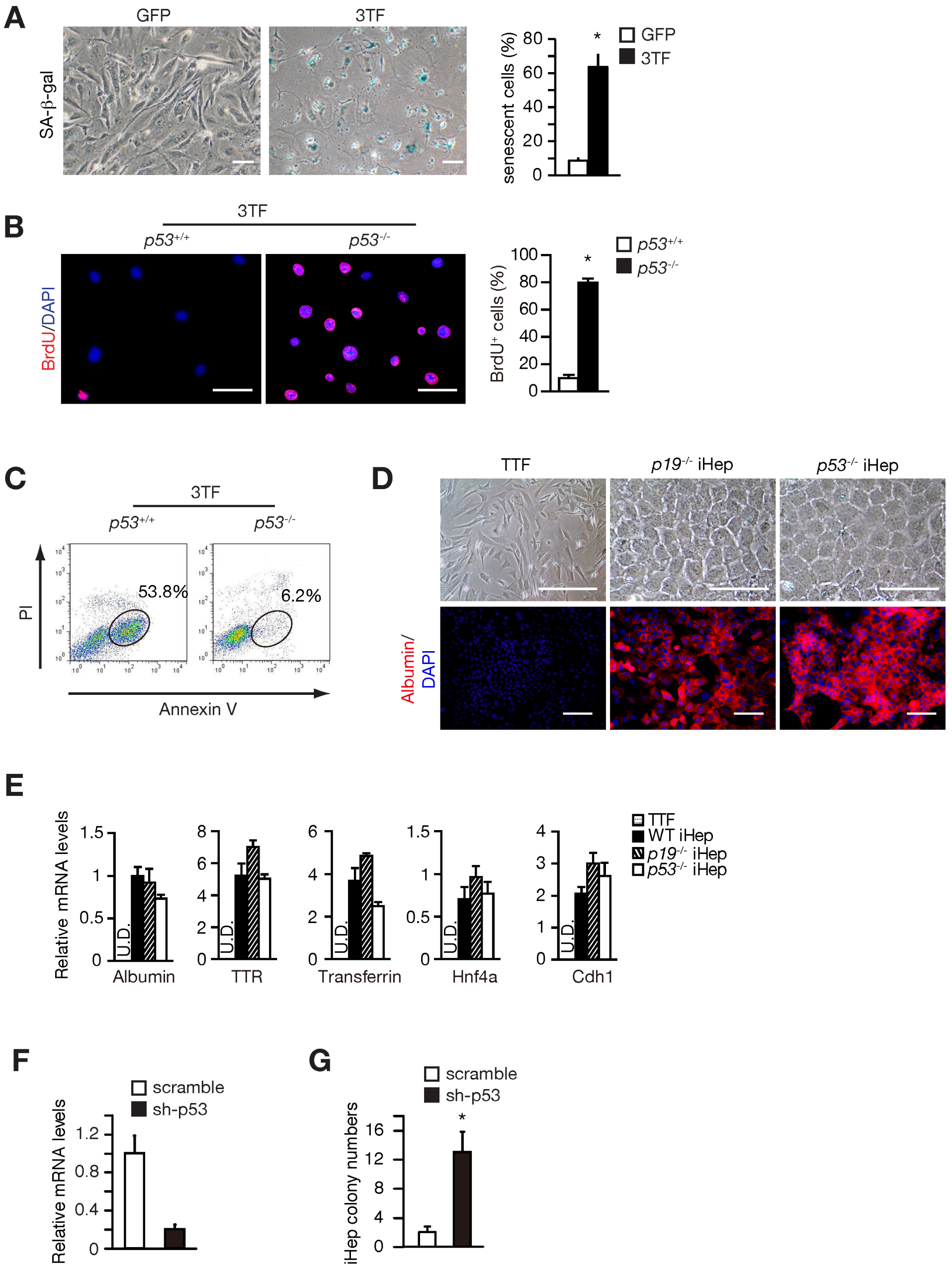

Supplementary information, Figure S1 Inhibition of p53 enhances iHep formation. (**A**) 3TF-induced cellular senescence in wild-type (WT) tail-tip fibroblasts (TTFs) was determined by β-galactosidase (β-gal) staining at day 6 after 3TF transduction. β-gal positive cells were quantified (*n*=4 fields). (**B** and **C**) 3TF-induced proliferation arrest (B) and cell death (C) in *p53*$^{-/-}$ cells were measured by BrdU incorporation and staining at

day 3 (B) and Annexin V-PI staining and flow cell sorting at day 6 (C), *n*=7 fields. (**D**) Immunofluorescent staining of Albumin in WT, *p19*$^{-/-}$ and *p53*$^{-/-}$ iHep cells. (**E**) qRT-PCR analyses of hepatic gene expression in WT, *p19*$^{-/-}$ and *p53*$^{-/-}$ iHep cells. Expression levels were normalized to those in livers. *Actin* was used as the reference gene. (**F**) shRNA-mediated p53 knockdown efficiency was examined by qRT-PCR assay. **(G**) iHep cell colonies were counted at day 8 after induction of hepatic conversion (*n*=4 independent experiments). Error bars indicate s.d.. *: *P*<0.05. Student's *t*-test.
