# Supplementary material for: Baf60b-mediated ATM-p53 activation blocks cell identity conversion by sensing chromatin opening
Source: Cell Res. 2017 Mar 17;27(5):642–56. doi: 10.1038/cr.2017.36 (PMC5520852; doi:10.1038/cr.2017.36)
Supplement: Supplementary information, Figure S2 — Unchanged levels of oncogenes and ROS during 3TF-induced hepatic conversion. [file cr201736x2.pdf]

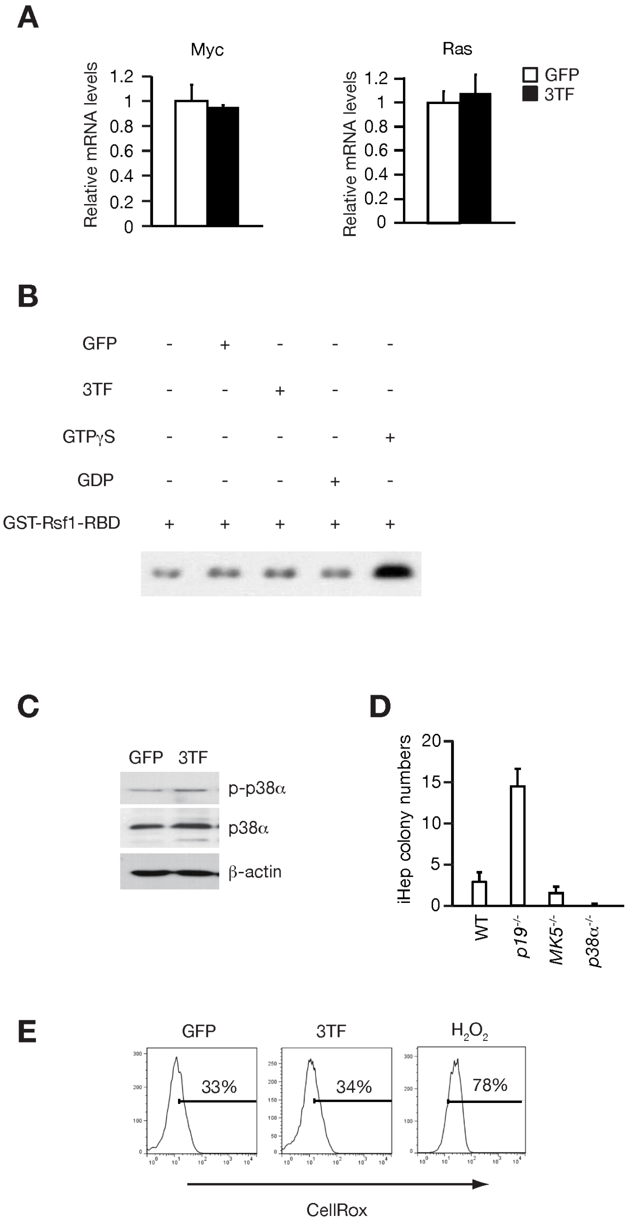

Supplementary information, Figure S2 Unchanged levels of oncogenes and ROS during 3TF-induced hepatic conversion. (**A**) Myc and Ras mRNA levels were characterized by qRT-PCR 48 hours after 3TF transduction. (**B**) The active Ras was pulled-down with Ras-binding domain (RBD) of Raf1 and immunoblotted with anti-Ras antibody. GTPγs and GDP were used to activate or inactivate Ras, that were used as

positive and negative controls. (**C**) Western blot analyses of p-p38α and p38α in 3TF-transduced WT TTFs. (**D**) iHep colony numbers were counted at day 8 after 3TF transduction in WT, *MK5*$^{-/-}$ and *p38α*$^{-/-}$ TTFs. MK5 and p38α are p53 upstream activators. (**E**) Cellular reactive oxygen species (ROS) levels was measured by CellROX^{TM} 48 hours after 3TF transduction and analyzed by flow cytometry. H_2O_2 treated cells were used as a control.
