# Supplementary material for: Baf60b-mediated ATM-p53 activation blocks cell identity conversion by sensing chromatin opening
Source: Cell Res. 2017 Mar 17;27(5):642–56. doi: 10.1038/cr.2017.36 (PMC5520852; doi:10.1038/cr.2017.36)
Supplement: Supplementary information, Figure S3 — ATM inactivation facilitates iHep formation. [file cr201736x3.pdf]

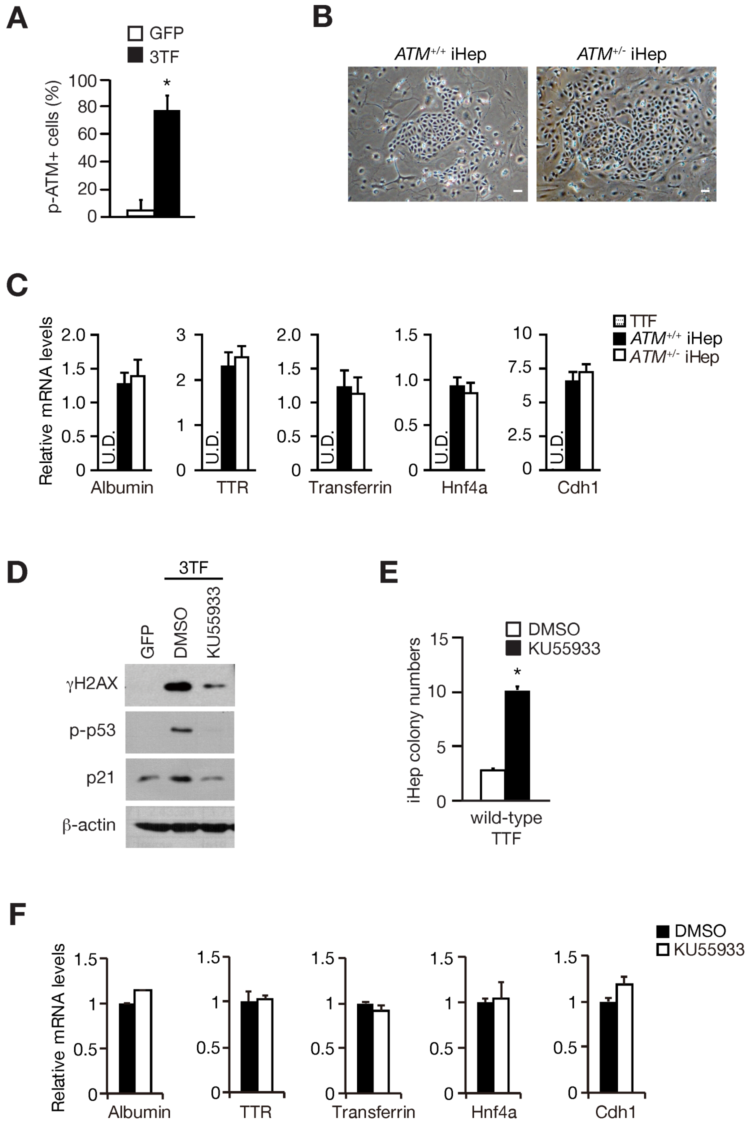

Supplementary information, Figure S3 ATM inactivation facilitates iHep formation. (**A**) p-ATM-positive cell numbers were quantified and presented as percentage. Cells showed >3 p-ATM foci in the nucleus were defined as p-ATM-positive cells. (**B**) Both *ATM*$^{+/-}$ and *ATM*$^{+/+}$ iHep cells displayed epithelial morphology. (**C**) qRT-PCR analyses of hepatic gene expression in *ATM*$^{+/+}$ and *ATM*$^{+/-}$ iHep cells. Expression levels were normalized to those in livers. *Actin* was used as the reference gene. (**D-F**) Treatment with ATM inhibitor KU55933 (10 μM from day 1 to day 3) decreased γH2AX and p-p53

levels after 3TF transduction as determined by western blotting (D). KU55933 treatment improved iHep formation (E). n=3 independent experiments. The induced expression of the hepatic genes was not affected by KU55933 (F). Error bars indicate s.d.. *: $P<0.05$. Student's *t*-test.
