# Supplementary material for: Baf60b-mediated ATM-p53 activation blocks cell identity conversion by sensing chromatin opening
Source: Cell Res. 2017 Mar 17;27(5):642–56. doi: 10.1038/cr.2017.36 (PMC5520852; doi:10.1038/cr.2017.36)
Supplement: Supplementary information, Figure S4 — DNA damage is not detected in hepatic conversion. [file cr201736x4.pdf]

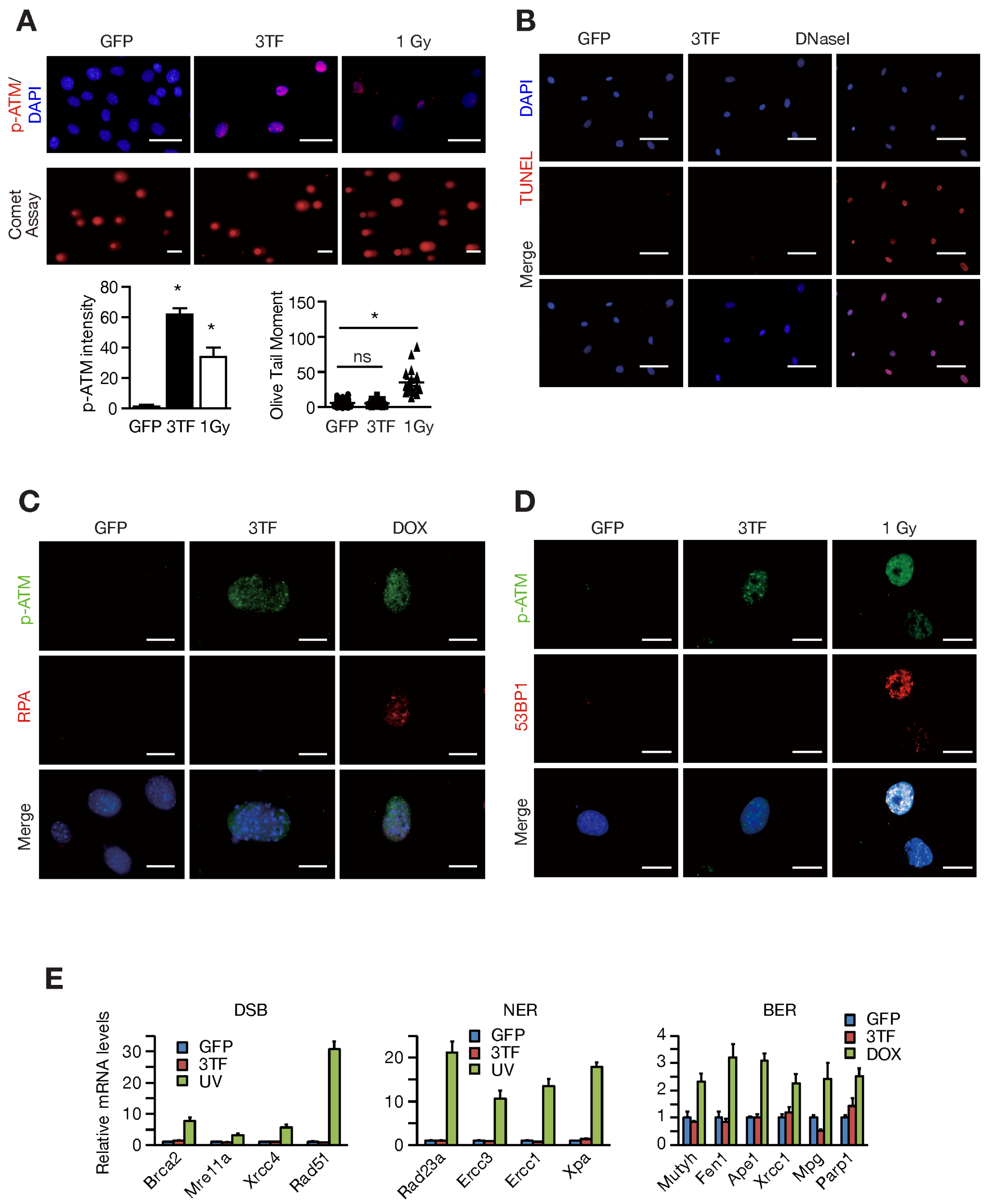

Supplementary information, Figure S4 DNA damage is not detected in hepatic conversion. (**A**) DNA strand breaks were analyzed by alkaline comet assay. Compared to low dose γ-irradiation (1Gy), DNA strand breaks were not detected after 3TF transduction. p-ATM fluorescent intensity was quantified by LAS AF Lite software. Olive tail moment was calculated by CASP. *n*=50 cells for GFP and 3TF groups, *n*=24

cells for 1Gy γ-irradiation group. (**B**) DNA fraction during hepatic conversion was measured by the TUNEL assay. DNaseI treatment was used as positive control. (**C** and **D**) RPA activation (C) and 53BP1 (D) activation were measured by immunofluorescent staining 48 hours after 3TF transduction. Doxorubicin treatment (C) and γ-irradiation (D) were used as positive controls. (**E**) qRT-PCR analyses of expression levels of genes involved in double strand break (DSB) repair, nucleotide excision repair (NER) and base excision repair (BER). Error bars indicate s.d.. *: $P<0.05$. Student's *t*-test.
