# Supplementary material for: Baf60b-mediated ATM-p53 activation blocks cell identity conversion by sensing chromatin opening
Source: Cell Res. 2017 Mar 17;27(5):642–56. doi: 10.1038/cr.2017.36 (PMC5520852; doi:10.1038/cr.2017.36)
Supplement: Supplementary information, Figure S5 — ATMIN controls 3TF-induced ATM activation. [file cr201736x5.pdf]

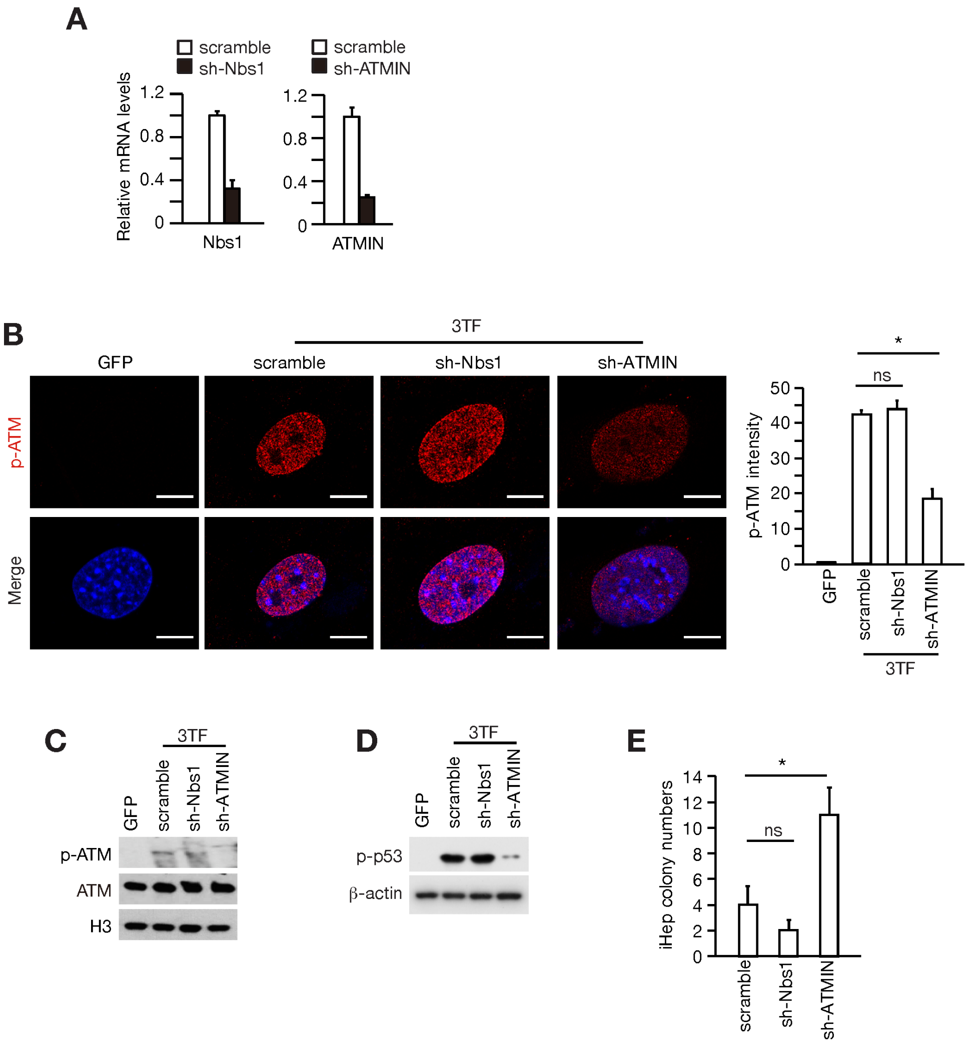

Supplementary information, Figure S5 ATMIN controls 3TF-induced ATM activation. (**A**) shRNA-mediated Nbs1 (sh-Nbs1) and ATMIN (sh-ATMIN) knockdown efficiency was examined by qRT-PCR. (**B**) Immunostaining of p-ATM in sh-Nbs1 and sh-ATMIN TTFs after 3TF transduction. p-ATM fluorescent intensity was quantified by LAS AF Lite software. *n*=26 cells. (**C-D**) p-ATM and p-p53 levels were measured by western blotting in 3TF-transduced TTFs with Nbs1 or ATMIN knockdown. (**E**) Numbers of iHep colonies were quantified at day 8 after 3TF transduction. *n*=4 independent experiments. Error bars indicate s.d.. *: $P<0.05$. Student's *t*-test.
