# Supplementary material for: Baf60b-mediated ATM-p53 activation blocks cell identity conversion by sensing chromatin opening
Source: Cell Res. 2017 Mar 17;27(5):642–56. doi: 10.1038/cr.2017.36 (PMC5520852; doi:10.1038/cr.2017.36)
Supplement: Supplementary information, Figure S6 — Analyses of upstream regulatory regions of Albumin and Hnf4α genes. [file cr201736x6.pdf]

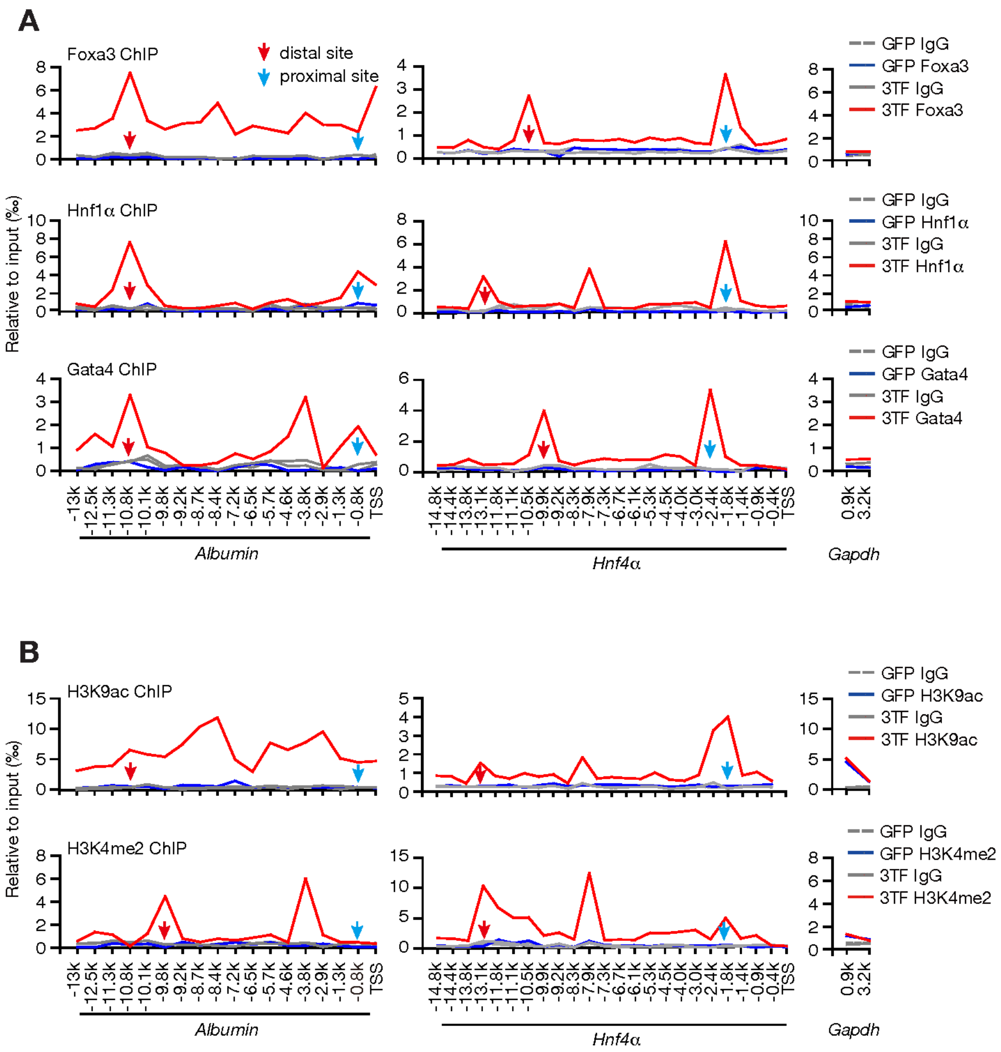

Supplementary information, Figure S6 Analyses of upstream regulatory regions of *Albumin* and *Hnf4α* genes. 13kb upstream of transcription starting site (TSS) of the *Albumin* gene and 14.8kb upstream of the *Hnf4α* TSS were analyzed. The binding of the 3TF to these two regions (**A**) and active histone marks, H3K9ac and H3K4me2, (**B**) were determined by the ChIP-qPCR assay. The promoter region of the *Gapdh* gene was used as a control in these analyses. Red and blue arrows indicate the distal site and proximal site, respectively, which were characterized in the time-course experiments in Figure 2.
