# Supplementary material for: Baf60b-mediated ATM-p53 activation blocks cell identity conversion by sensing chromatin opening
Source: Cell Res. 2017 Mar 17;27(5):642–56. doi: 10.1038/cr.2017.36 (PMC5520852; doi:10.1038/cr.2017.36)
Supplement: Supplementary information, Figure S7 — Time-course analyses of ATM and p53 activation during hepatic conversion. [file cr201736x7.pdf]

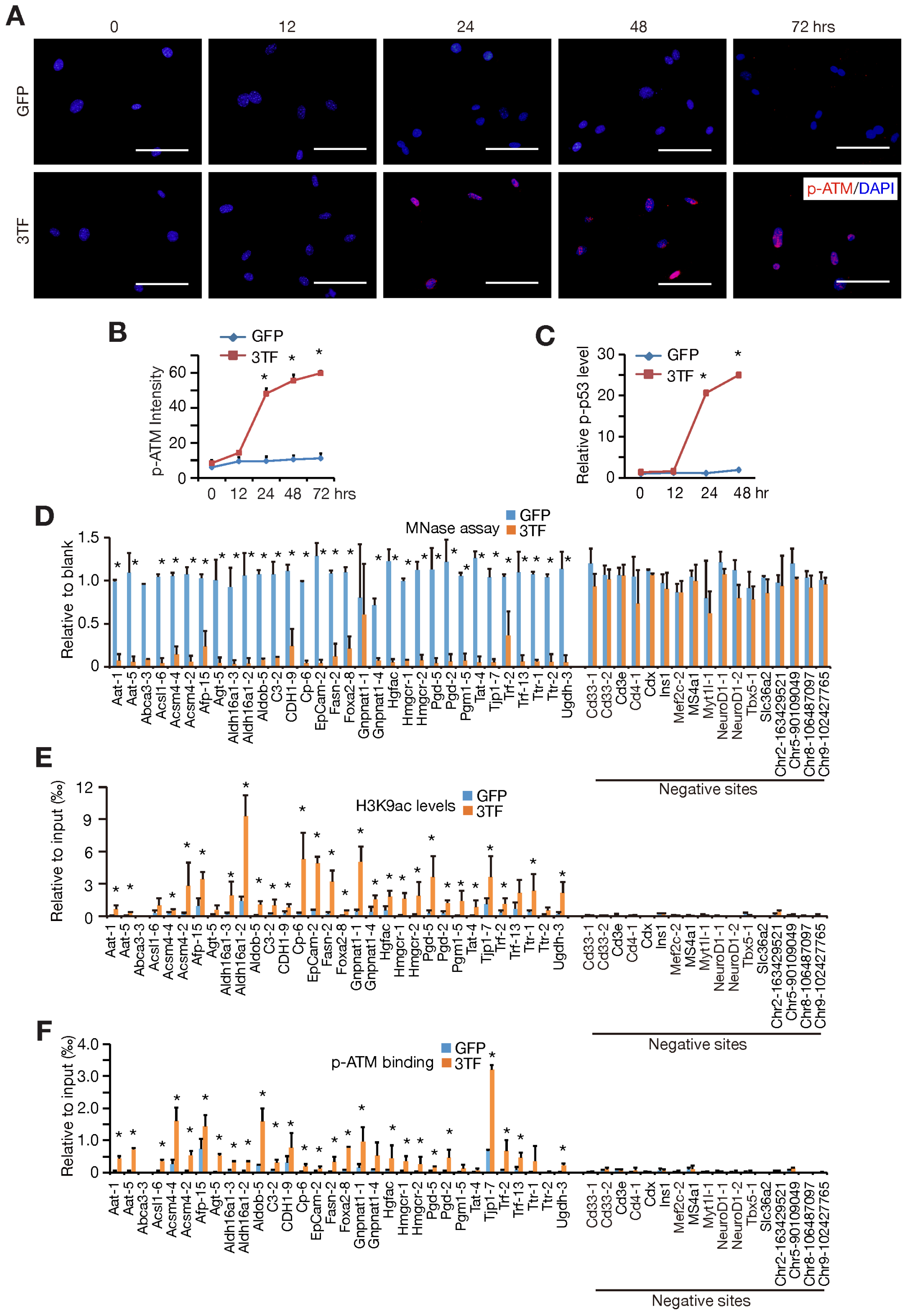

Supplementary information, Figure S7 Time-course analyses of ATM and p53 activation during hepatic conversion. (**A**) The activation of ATM was analyzed by

immunofluorescent staining of p-ATM at different time points. (**B**) The p-ATM intensity was quantified using LAS AF Lite. n=6 cells for each time point. (**C**) The p-p53 levels were determined by western blotting in Fig. 2G and quantified by Image J. Error bars indicate s.d.. *: $P<0.05$. Student's t-test. (**D-E**) Chromatin opening and active histone mark of H3K9ac at a panel of hepatic gene loci. (**F**) Binding of p-ATM to a panel of hepatic gene loci were analyzed by the ChIP-qPCR assay at 48 hours after 3TF transduction. For D-F, we compared ChIP-qPCR data between the control (GFP) and experiment groups (3TF). Regarding negative sites, we analyzed 4 intergenic loci and 15 loci at genes specifically expressed in other types of terminally differentiated cells. Data represent 3 independent experiments. Error bars indicate s.d.. *: $P<0.05$, student's t-test. Original ChIP-qPCR data were provided in Table S3.
