# Supplementary material for: Baf60b-mediated ATM-p53 activation blocks cell identity conversion by sensing chromatin opening
Source: Cell Res. 2017 Mar 17;27(5):642–56. doi: 10.1038/cr.2017.36 (PMC5520852; doi:10.1038/cr.2017.36)
Supplement: Supplementary information, Figure S8 — Validation of Baf60b in controlling ATM activation. [file cr201736x8.pdf]

A

Family	Gene Symbol	Gene ID	shRNA K.D. Efficiency
SWI/SNF	Brg1	20586	94%
	Brm	67155	89%
	Baf250	93760	65%
	Baf170	68094	70%
	Baf155	20588	62%
	Baf60a	83797	60%
	Baf60b	83796	94%
	Baf60c	66993	98%
CHD	CHD1	12684	81%
	CHD3	21684	71%
	CHD4	107932	84%
	MBD2	17191	73%
	MTA1	116870	58%
	MTA2	23942	76%
	MTA3	116871	60%
	HDAC2	15182	89%
	GATAD2A	24366	63%
INO80	P400	75560	66%
	H2AZ	51788	75%
	p120	12388	79%
	Tip60	81601	77%
	Arp5	109275	82%
	Arp6	67019	91%
	Arp8	56249	59%
	RUVBL2	20174	94%
	Gas41	64050	66%
	Dmap1	66233	93%
	Brd8	78656	67%
	Mrg15	21761	91%
	Mrgx	56397	92%
ISWI	SNF2L	93761	91%
	SNF2H	93762	77%
	BPTF	207165	78%
	WCRF180	217578	81%
	RbAp46	245688	84%
	RbAp48	19646	62%
Relevant Proteins	Brit1	244329	86%
	KAP1	21849	90%
	Kat8	67773	82%

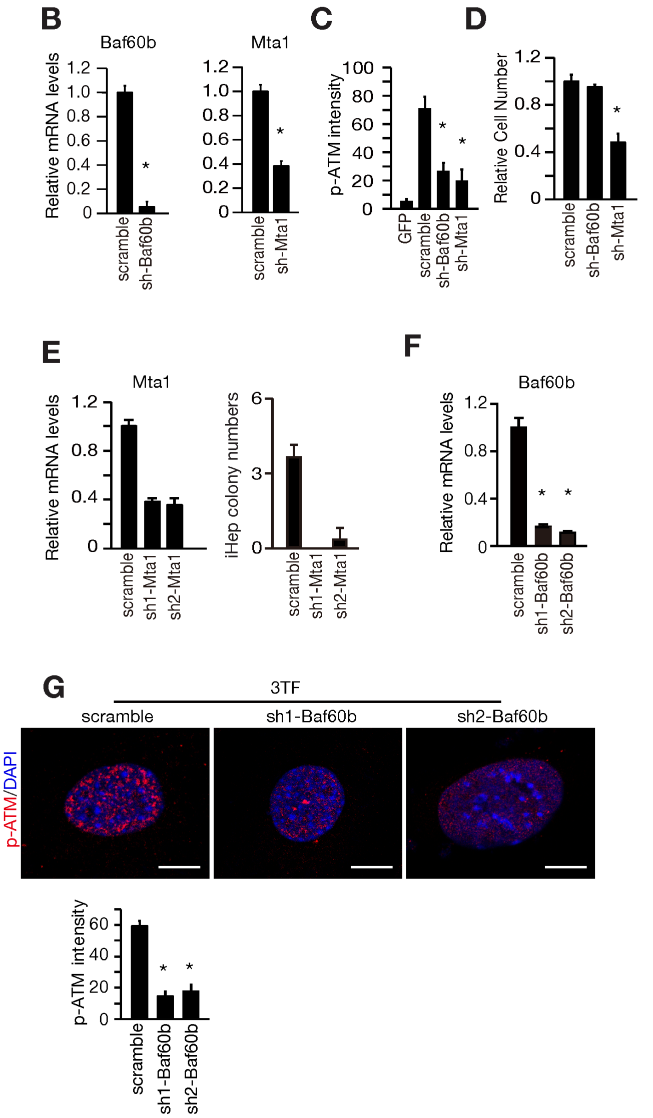

Supplementary information, Figure S8 Validation of Baf60b in controlling ATM activation. (**A**) Chromatin remodeler complex proteins screened for 3TF-induced ATM activation. (**B**) Knockdown efficiencies of shRNAs against Baf60b (sh-Baf60b) and Mta1 (sh-Mta1) were determined by qRT-PCR. (**C**) sh-Baf60b and sh-Mta1 caused significant reduced p-ATM levels as determined by immunofluorescent staining, *n*=16 cells for each group. (**D**) sh-Mta1, but not sh-Baf60b, led to decreased number of TTF in culture. Same numbers of cells were seeded and total cell numbers were quantified 3 days after seeding.

n=3 for each group. (**E**) Knockdown efficiencies of shRNAs against Mta1 were determined by qRT-PCR. iHep colony numbers were quantified at day 8 after 3TF transduction. *n*=3 independent experiments for each group. (**F**) Knockdown efficiency of two additional shRNAs against Baf60b as determined by qRT-PCR. Data represent two independent experiments. (**G**) p-ATM levels were analyzed by immunofluorescent staining and quantified by LAS AF Lite. *n*=19 cells for each group. Scale bar: 10 μm. Error bars indicate s.d.. *: $P<0.05$. Student's *t*-test.
