# Supplementary material for: Baf60b-mediated ATM-p53 activation blocks cell identity conversion by sensing chromatin opening
Source: Cell Res. 2017 Mar 17;27(5):642–56. doi: 10.1038/cr.2017.36 (PMC5520852; doi:10.1038/cr.2017.36)
Supplement: Supplementary information, Figure S9 — Baf60b inhibition facilitates iHep formation. [file cr201736x9.pdf]

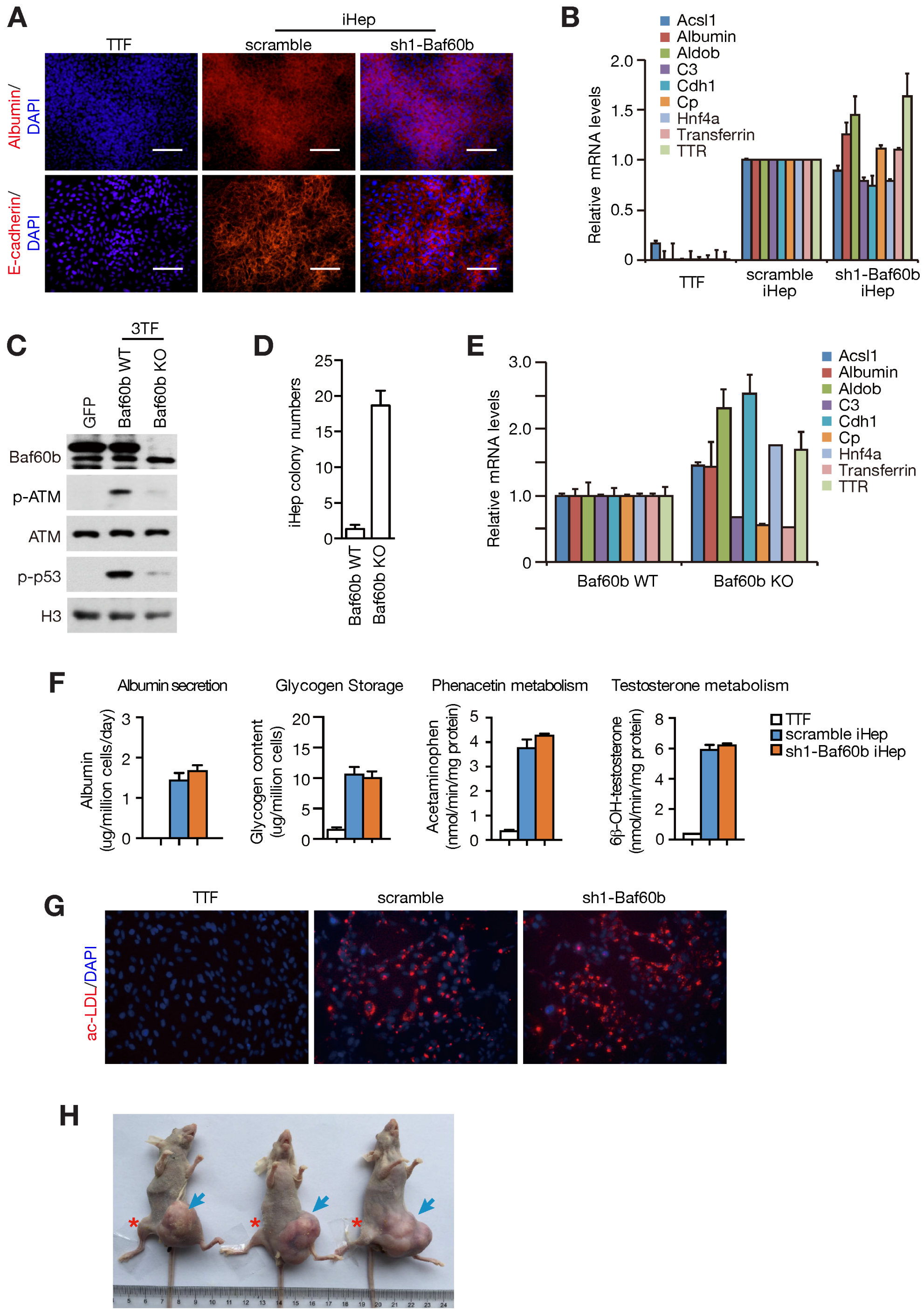

Supplementary information, Figure S9 Baf60b inhibition facilitates iHep formation. (**A-B**) sh-Baf60b iHep cells characterization. Immunofluorescent staining of albumin and E-cadherin in sh1-Baf60b and scramble control iHep cells (**A**). qRT-PCR analyses of

hepatic gene expression in Baf60b-knockdown (sh1-Baf60b) and scramble control iHep cells (**B**). Expression levels were normalized to those in livers. *Actin* was used as the reference gene. (**C**) p-ATM and p-p53 levels during hepatic conversion were determined by western blotting in Baf60b knockout cells. (**D**) iHep colony numbers were quantified at day 8 after 3TF transduction. *n*=4 independent experiments for each group. Error bars indicate s.d.. (**E**) qRT-PCR analyses of hepatic gene expression in Baf60b-knockout (Baf60b-KO) and Baf60b WT iHep cells. (**F-G**) Functional characterization of Baf60b-knockdown iHep cells. Albumin excretion, glycogen storage, CYP metabolic activities were measured in Baf60b-KO iHep cells (F). The metabolic products of phenacetin (converted to acetaminophen by Cyp1a2), testosterone (converted to 6b-OH-testosterone by Cyp3a enzymes) were determined by liquid chromatograph-tandem mass spectrometry according to standard curve. Lipid intake was determined using fluorescent group labeled acetylated LDL (G). (**H**) $3x10^6$ sh1-Baf60b iHep cells (red asteroids) and human liver cancer cell SNU398 (blue arrows) were subcutaneously transplanted into the flanks of each nude mouse, respectively. SNU398-generated tumors are indicated by the arrows.
