# Supplementary material for: Baf60b-mediated ATM-p53 activation blocks cell identity conversion by sensing chromatin opening
Source: Cell Res. 2017 Mar 17;27(5):642–56. doi: 10.1038/cr.2017.36 (PMC5520852; doi:10.1038/cr.2017.36)
Supplement: Supplementary information, Figure S10 — Baf60b recruited ATM and p53 to the SWI/SNF complex. [file cr201736x10.pdf]

A

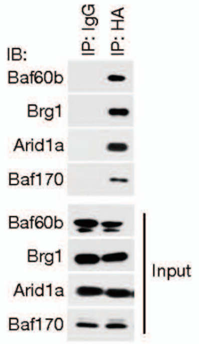

B

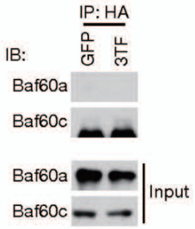

C

Protein ID	Gene names	Normalized LFQ intensity		
		GFP	3TF	3TF ΔSWIB
Q3TKT4	Brg1 (Smarca4)	5.933E+08	8.274E+08	0
F2Z4A9	Brm (Smarca2)	1.091E+07	0	0
E9QAQ7	Baf250a (Arid1a)	2.461E+08	6.063E+07	0
E9Q6R4	Baf250b (Arid1b)	8.574E+07	4.120E+07	0
Q3UNN4	Baf155 (Smarcc1)	4.241E+08	6.721E+08	0
Q3UID0	Baf170 (Smarcc2)	1.254E+09	2.640E+09	0
Q61466	Baf60a (Smarcd1)	UD	UD	UD
Q99JR8	Baf60b (Smarcd2)	1.758E+09	1.758E+09	1.758E+09
Q6P9Z1	Baf60c (Smarcd3)	UD	UD	UD
O54941	Baf57 (Smarce1)	1.115E+09	1.563E+09	5.191E+08
Q9Z2N8	Baf53a (Actl6a)	2.277E+08	6.625E+08	2.170E+08
Q9Z0H3	Baf47 (Smarcb1)	2.770E+08	1.389E+08	0
K4DI61	Baf45a (Phf10)	1.177E+07	3.368E+07	0
Q62388	Atm	0	4.603E+06	0
I7HIK9	Trp53 (p53)	0	8.108E+06	0

Supplementary information, Figure S10 Baf60b recruited ATM and p53 to the SWI/SNF complex. (**A**) TTFs were transduced with HA-tagged Baf60b. Cell lysates were immunoprecipitated (IP) with an HA antibody or control IgG followed by the immunoblotting (IB) assay with antibodies against indicated proteins. (**B**) 48 hours after 3TF transduction, cell lysates were immunoprecipitated (IP) with an HA antibody or control IgG followed by immunoblot (IB) assays with antibodies against indicated proteins. (**C**) List of Baf60b-interacted proteins identified and quantified by mass spectrometry. Label-Free-Quantification (LFQ) was used to quantify the related protein intensity. GFP: TTF transduced with GFP control at 48 hours. 3TF: TTF transduced with

3TF at 48 hours. ΔSWIB: TTF transduced with 3TF and a Baf60b mutant lacking SWIB domain. Original data were shown in Table S10.
