# Supplementary material for: Baf60b-mediated ATM-p53 activation blocks cell identity conversion by sensing chromatin opening
Source: Cell Res. 2017 Mar 17;27(5):642–56. doi: 10.1038/cr.2017.36 (PMC5520852; doi:10.1038/cr.2017.36)
Supplement: Supplementary information, Figure S11 — Analyses of the binding of the SWI/SNF complex to hepatic gene loci. [file cr201736x11.pdf]

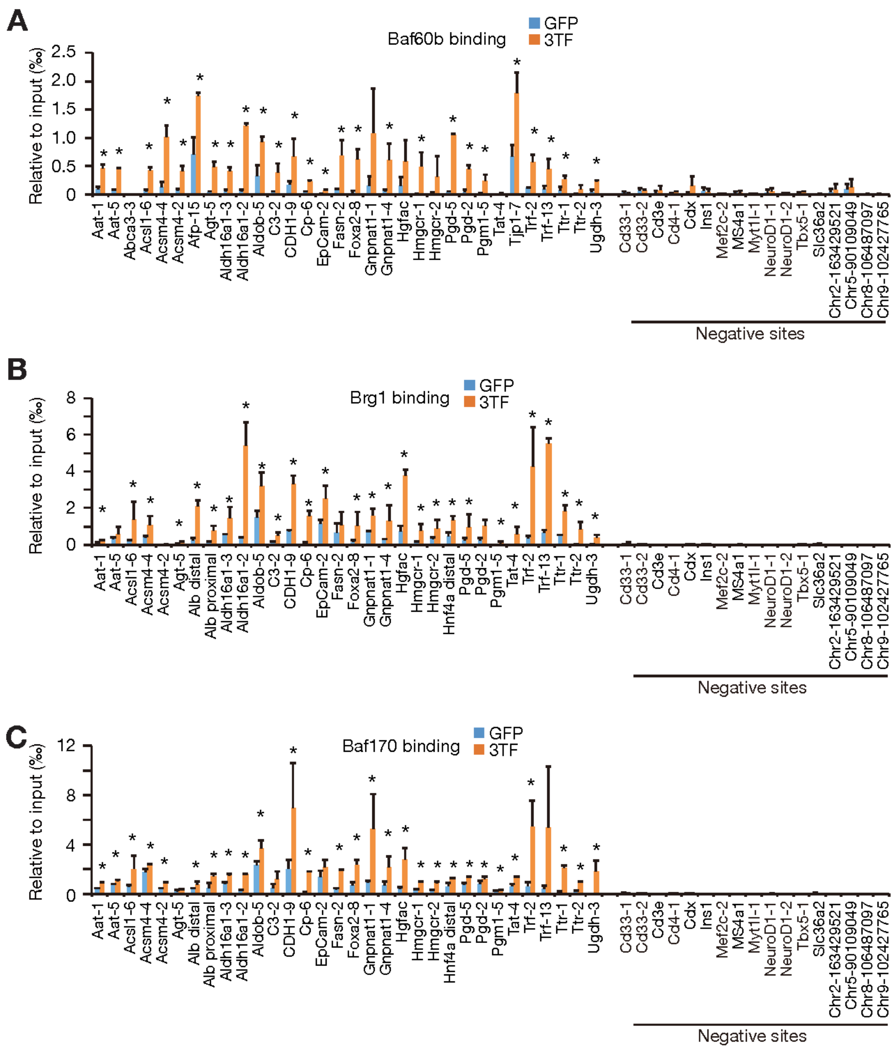


**Supplementary information, Figure S11** Analyses of the binding of the SWI/SNF complex to hepatic gene loci. (**A**) The binding of Baf60b at hepatic gene loci was determined at 48 hours after 3TF transduction by the ChIP-qPCR assay. For negative sites, we analyzed 4 intergenic loci and 15 loci at genes specifically expressed in other types of terminally differentiated cells. Data represent 3 independent experiments. Error bars indicate s.d.. *: $P<0.05$, student's *t*-test. Original ChIP-qPCR data were provided in Table S3. (**B**) The binding of the SWI/SNF complex members Brg1 and Baf170 to

hepatic gene loci was analyzed by the ChIP-qPCR assay at 48 hours after 3TF transduction. Data represent 3 independent experiments. Error bars indicate s.d.. *: $P<0.05$, student's *t*-test. Original ChIP-qPCR data were provided in Table S4.
