# Supplementary material for: Baf60b-mediated ATM-p53 activation blocks cell identity conversion by sensing chromatin opening
Source: Cell Res. 2017 Mar 17;27(5):642–56. doi: 10.1038/cr.2017.36 (PMC5520852; doi:10.1038/cr.2017.36)
Supplement: Supplementary information, Figure S12 — Chromatin opening by Brg1 and Baf60b. [file cr201736x12.pdf]

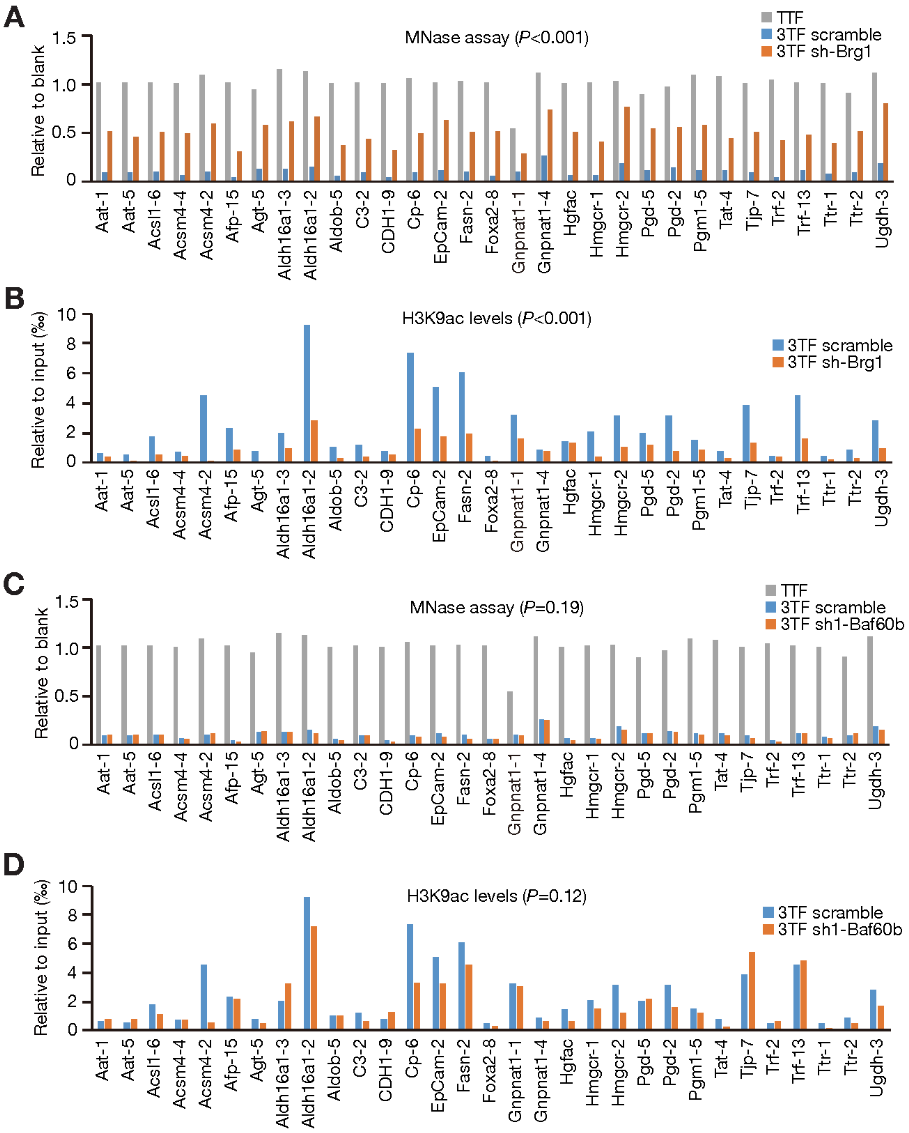

Supplementary information, Figure S12 Chromatin opening by Brg1 and Baf60b**.** Brg1 knockdown and Baf60b knockdown TTFs were induced hepatic conversion by 3TF transduction. 48 hours after 3TF transduction, chromatin opening was measured by MNase assay on hepatic gene loci (**A, C**), and active mark H3K9ac was determined using ChIP-qPCR (**B, D**). Scramble shRNA was used as control. ChIP-qPCR data between the two groups (scramble vs. shRNA) were compared. Student's *t*-test was applied. *P* values are provided. Original ChIP-qPCR data were available in Table S5.
