# Supplementary material for: Baf60b-mediated ATM-p53 activation blocks cell identity conversion by sensing chromatin opening
Source: Cell Res. 2017 Mar 17;27(5):642–56. doi: 10.1038/cr.2017.36 (PMC5520852; doi:10.1038/cr.2017.36)
Supplement: Supplementary information, Figure S13 — Baf60a and Baf60c replace the chromatin-remodeling function of Baf60b in Baf60b-deficient cells. [file cr201736x13.pdf]

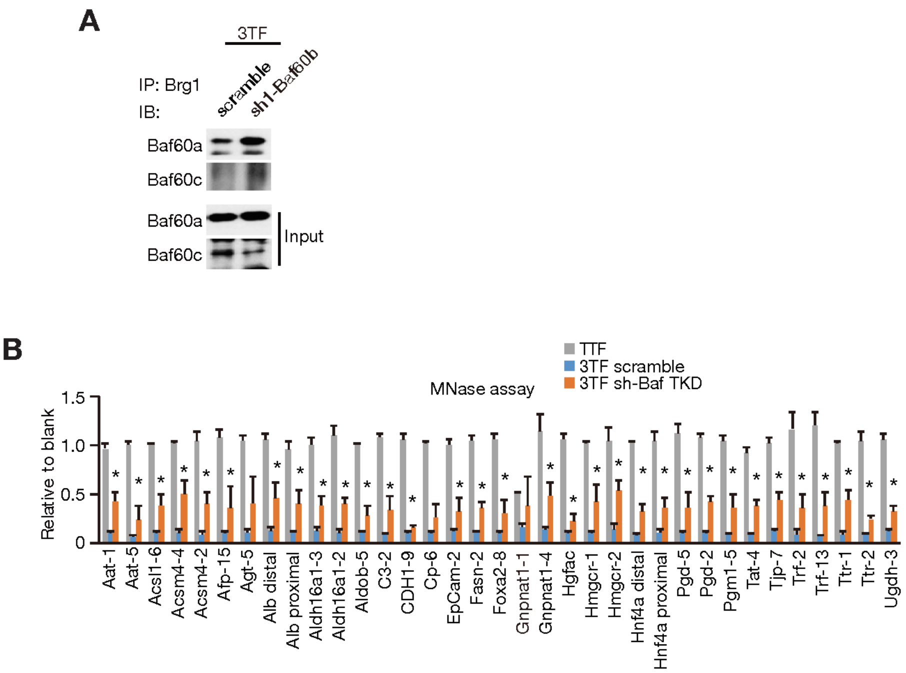

Supplementary information, Figure S13 Baf60a and Baf60c replace the chromatin-remodeling function of Baf60b in Baf60b-deficient cells. (**A**) TTFs transfected with sh1-Baf60b were induced hepatic conversion by 3TF. 48 hours after 3TF transduction, cell lysates were immunoprecipitated (IP) with Brg1 antibody followed by immunoblot assays (IB) using Baf60a and Baf60c antibodies. (**B**) TTFs transfected with shRNAs targeting Baf60a, Baf60b and Baf60c together (TKD) were induced hepatic conversion by 3TF. 48 hours after 3TF transduction, chromatin opening was measured using MNase assay. Scramble shRNA transfection was used as control. ChIP-qPCR data between the two groups (scramble vs. shRNA) were compared. Student's *t*-test was applied. Data represent 3 independent experiments. Error bars indicate s.d.. *: $P<0.05$, student's *t*-test. Original ChIP-qPCR data were available in Table S6.
