# Supplementary material for: Baf60b-mediated ATM-p53 activation blocks cell identity conversion by sensing chromatin opening
Source: Cell Res. 2017 Mar 17;27(5):642–56. doi: 10.1038/cr.2017.36 (PMC5520852; doi:10.1038/cr.2017.36)
Supplement: Supplementary information, Figure S14 — Baf60b mediates ATM recruitment. [file cr201736x14.pdf]

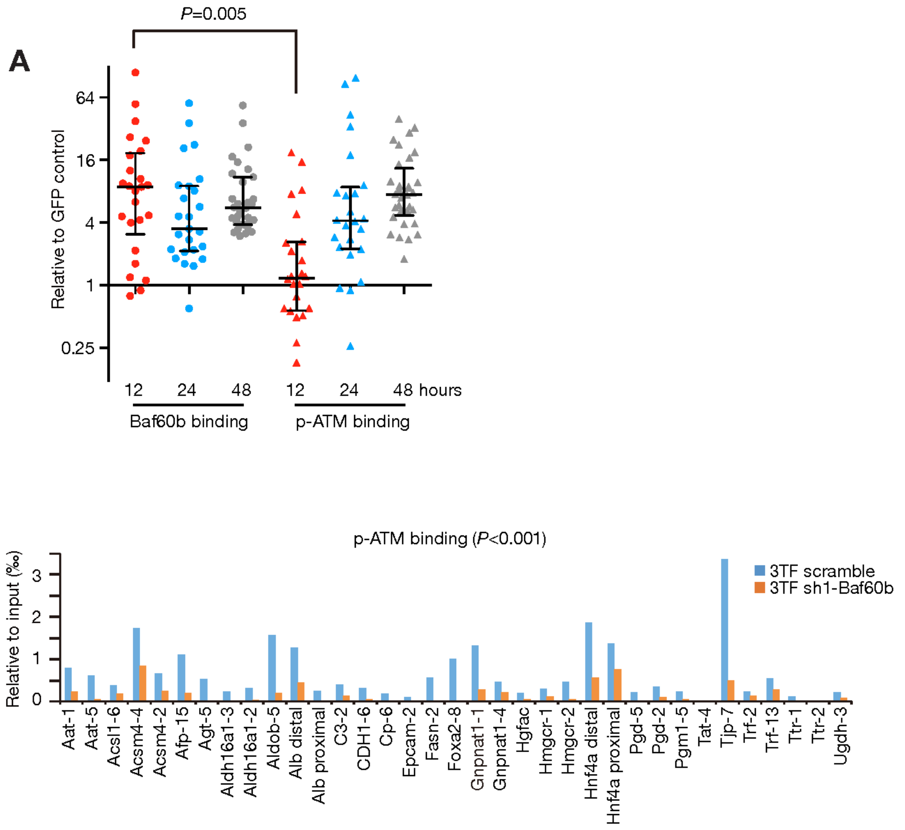

Supplementary information, Figure S14 Baf60b mediates ATM recruitment. (**A**) TTFs were collected at 12, 24, and 48 hours after 3TF transfection. Bindings of Baf60b and p-ATM at hepatic gene loci were determined by ChIP-qPCR and normalized to data in GFP controls. Student's *t*-test was applied. Original ChIP-qPCR data were available in Table S7 and S3. (**B**) TTFs transfected with sh1-Baf60b were induced hepatic conversion by 3TF. 48 hours after 3TF transduction, p-ATM binding was measured by ChIP-qPCR. Scramble shRNA transfection was used as control. ChIP-qPCR data between the two groups were compared. Student's *t*-test was applied. Original ChIP-qPCR data were available in Table S8.
