# Supplementary material for: Baf60b-mediated ATM-p53 activation blocks cell identity conversion by sensing chromatin opening
Source: Cell Res. 2017 Mar 17;27(5):642–56. doi: 10.1038/cr.2017.36 (PMC5520852; doi:10.1038/cr.2017.36)
Supplement: Supplementary information, Figure S15 — ATMIN is responsible for phosphorylation of Baf60b-recruited ATM. [file cr201736x15.pdf]

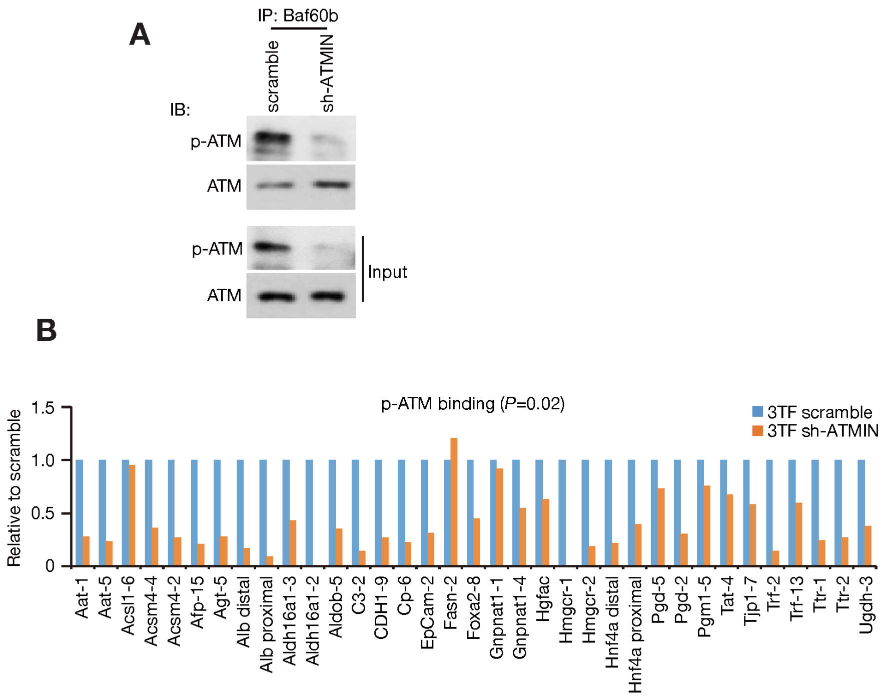

Supplementary information, Figure S15 ATMIN is responsible for phosphorylation of Baf60b-recruited ATM. (**A**) TTFs transfected with ATMIN shRNA were induced hepatic conversion by 3TF. 48 hours after 3TF transduction, cell lysates were immunoprecipitated with Baf60b antibody followed by immunoblot assays with p-ATM and ATM antibody. (**B**) p-ATM binding was measured by ChIP-qPCR. Scramble shRNA transfection was used as control. ChIP-qPCR data between the two groups were compared. Student's *t*-test was applied. Original ChIP-qPCR data were available in Table S9.
