# Supplementary material for: Baf60b-mediated ATM-p53 activation blocks cell identity conversion by sensing chromatin opening
Source: Cell Res. 2017 Mar 17;27(5):642–56. doi: 10.1038/cr.2017.36 (PMC5520852; doi:10.1038/cr.2017.36)
Supplement: Supplementary information, Figure S16 — Baf60b-mediated ATM recruitment facilitates ATM activation. [file cr201736x16.pdf]

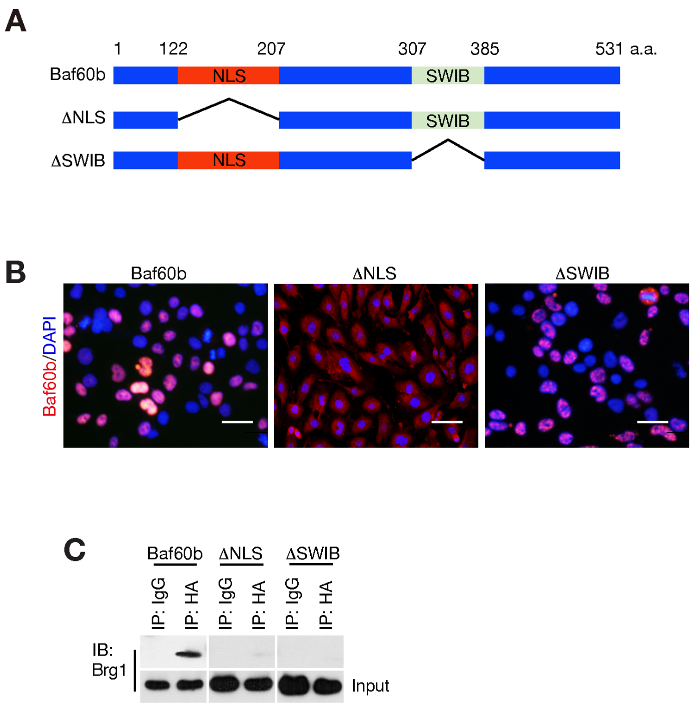

Supplementary information, Figure S16 Baf60b-mediated ATM recruitment facilitates ATM activation. (**A**) Two Baf60b mutants were cloned. One mutant deleted the nuclear location sequence (ΔNLS) and the other one deleted the conserved SWIB domain (ΔSWIB). Full length HA-tagged Baf60b was used as a control. All constructs were added the HA-tag at the N-terminal. (**B**) Immunostaining of HA-tag to determine the cellular localization of Baf60b mutants. (**C**) TTF cells were transfected with HA-tagged Baf60b and two Baf60b mutants. Cell lysates were immunoprecipitated (IP) with an HA antibody or control IgG followed by the immunoblotting (IB) assays with antibody against Brg1.
