# Supplementary material for: Baf60b-mediated ATM-p53 activation blocks cell identity conversion by sensing chromatin opening
Source: Cell Res. 2017 Mar 17;27(5):642–56. doi: 10.1038/cr.2017.36 (PMC5520852; doi:10.1038/cr.2017.36)
Supplement: Supplementary information, Figure S17 — Baf60b depletion facilitates iPS cell formation. [file cr201736x17.pdf]

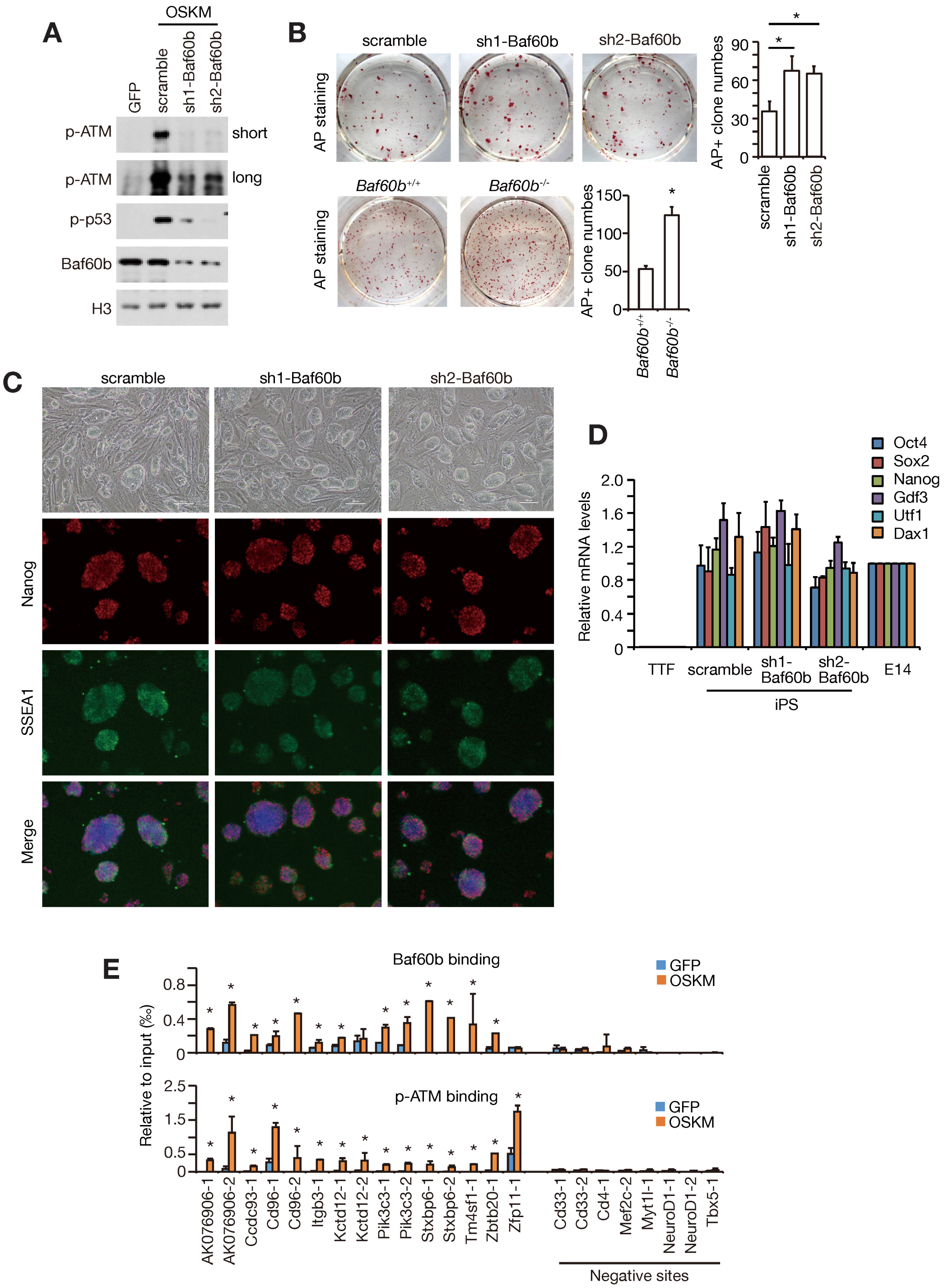

Supplementary information, Figure S17 Baf60b depletion facilitates iPS cell formation. (**A**) Baf60b-knockdown TTFs (sh1-Baf60b and sh2-Baf60b) were induced to pluripotent stem cell (iPSC) by Oct4, Sox2, Klf4 and c-Myc (OSKM). 48 hours after OSKM

transduction, p-ATM and p-p53 levels were measured by western blotting. (**B**) The induced iPS colonies were determined by AP staining in Baf60b-knockdown and Baf60b-knockout cells. iPS colonies were counted 14 days after OSKM transduction. (**C**) The morphology of iPS cells was documented, and pluripotent stem cell markers Nanog and SSEA1 were determined by immunofluorescent staining. (**D**) Expression levels of pluripotency genes were analyzed using qRT-PCR. Mouse E14 ES cells were used as positive control. Error bars indicate s.d.. (**E**) The bindings of Baf60b and p-ATM at OSKM-occupied gene loci were determined at 48 hours after OSKM transduction by the ChIP-qPCR assay. The OSKM-binding loci were selected according to published data (*10, 11*). Error bars indicate s.d.. *: $P<0.05$, student's *t*-test.
